# Tailoring C–H amination activity *via* modification of the triazole-derived carbene ligand†

**DOI:** 10.1039/d4dt01715c

**Published:** 2024-08-15

**Authors:** Luke A. Hudson, Wowa Stroek, Martin Albrecht

**Affiliations:** a Department of Chemistry, Biochemistry and Pharmaceutical Sciences, University of Bern Freiestrasse 3 3012 Bern Switzerland martin.albrecht@unibe.ch

## Abstract

Two new C,O-bidentate chelating triazolylidene-phenolate ligands were synthesized that feature a diisopropylphenyl (dipp) and an adamantyl (Ad) substituent respectively on the triazole scaffold. Subsequent metalation afforded iron(ii) complexes [Fe(C^O)_2_] that are active catalysts for the intramolecular C–H amination of organic azides. When compared to the parent complex containing a triazolylidene with a mesityl substituent (Mes) the increased steric bulk led to slightly lower activity (TOF_max_ = 23 h^−1^*vs.* 30 h^−1^), however selectivity towards pyrrolidine formation increases from 92% up to >99%. Kinetic studies indicate that the mechanism is similar in all three complexes and includes a half-order dependence in [Fe(C^O)_2_], congruent with the involvement of a dimetallic catalyst resting state within this catalyst class. Structural analysis suggests that enhanced bulkiness disfavors N_2_ loss and nitrene formation, yet shields the nitrene from intermolecular processes and thus favors intramolecular nitrene insertion into the C–H bond. This model rationalizes the high selectivity and the lower reaction rate observed with dipp and with Ad substituents on the ligand.

## Abstract 2

Tailoring of the wingtip groups of triazole-derived carbene ligands offers a rational approach for modulating the catalytic activity and selectivity of the iron center in C–H bond amination catalysis, and also reveals a general mechanistic profile.

## Graphical abstract

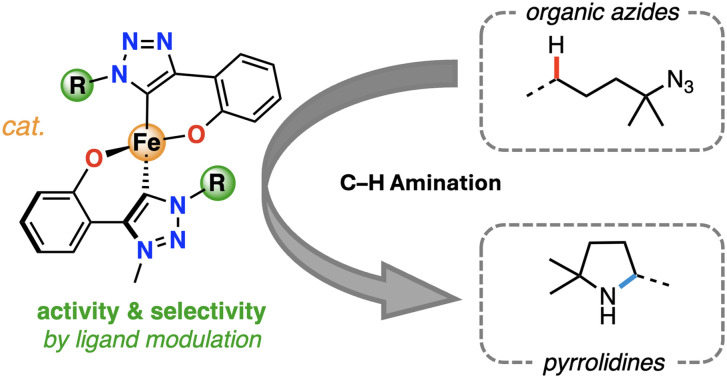

The direct amination of C–H bonds is an attractive method for constructing C–N bonds,^1^ as unlike classical methods,^2–4^ no carbon functionalization is required. Direct C–H amination relies on the availability of a nitrene as transient and active species, which is typically generated from azides upon N_2_ loss at a transition metal center.^1,5–11^ Iron complexes have been established as particularly active catalysts for this reaction, especially when using organic azides as nitrene precursors.^8,12–18^ Within this context, we recently demonstrated that complex 1 containing a triazole-derived carbene ligand is one of the best performing catalysts for C–H amination (Scheme 1), reaching record-high turnover numbers and state-of-the-art turnover frequencies.^19^

**Scheme 1:**
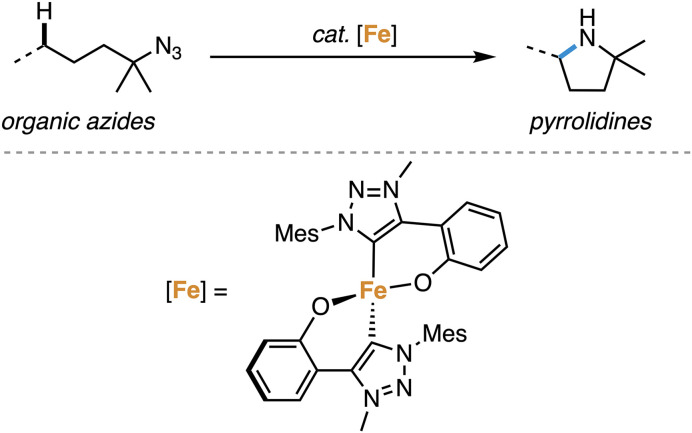
Application of mesoionic carbene iron complex 1 in intramolecular C–H amination catalysis.

Kinetic studies of this system revealed an unusual half-order rate dependence on iron catalyst concentration. Therefore, a mechanism was postulated that involves an off-cycle dimeric species as catalyst resting state that needs to be cleaved en-route to coordination of the azide and rate-limiting release of N_2_ to generate the reactive iron nitrene intermediate. Considering the synthetic flexibility of the triazole core of the ligand in complex 1,^20–23^ ligand tailoring provides a plausible strategy for modulating catalytic activity and selectivity.^24–28^ Specifically, we hypothesized that the use of bulkier wingtip groups on the carbene will destabilize the dimeric resting state and thus accelerate catalytic turnover.

Here, we demonstrate that substitution of the mesityl wingtip group of the carbenes in complex 1 for diisopropylphenyl (dipp) and adamantyl (Ad) substituents indeed affects the catalytic rates, whilst preserving the overall mechanism. Moreover, steric tailoring leads to enhanced chemoselectivity.

## Results and discussion

### Synthesis of the complexes

**Scheme 2:**
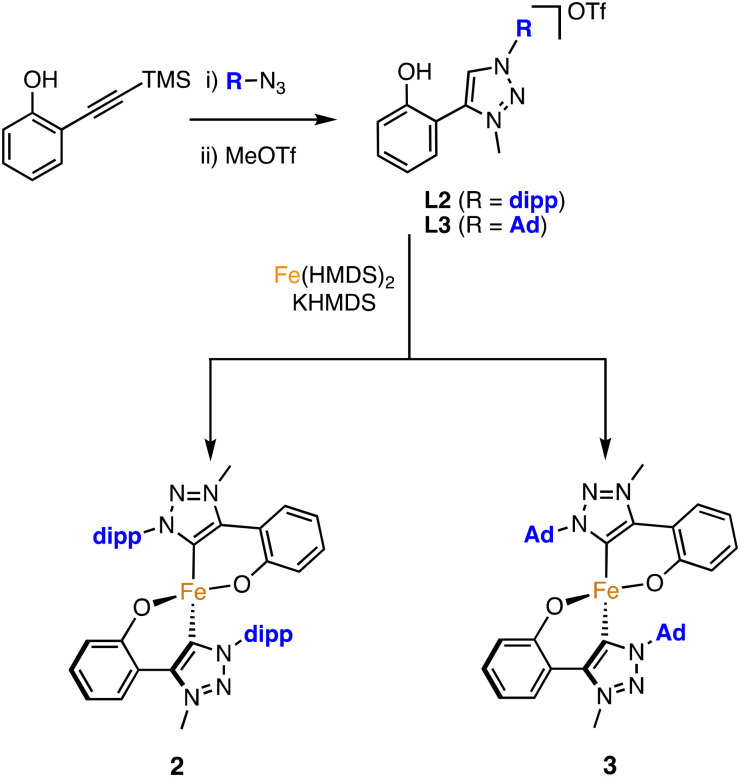
Synthesis of complexes 2 and 3.

The new ligand precursors L2 and L3 were synthesised according to a procedure adapted from the preparation of L1 (Scheme 2).^19^ Thus, “click”-type copper-catalysed azide–alkyne cycloaddition (CuAAC)^21,22,29^ of a silyl-protected alkynylphenol and the corresponding organic azide followed by methylation with methyl triflate (MeOTf) yielded L2 and L3 in good yields. Subsequent metalation with Fe(HMDS)_2_ in the presence of KHMDS afforded complexes 2 and 3 as highly air-sensitive, bright orange solids in 26% and 53% yield, respectively (HMDS = hexamethyldisilazide, N(SiMe_3_)_2_^−^).

Analytically pure complexes 2 and 3 were isolated from the reaction mixture after precipitation with *n*-hexane, subsequent washing of the solid residue with Et_2_O, and finally extraction into benzene followed by filtration. The ^1^H NMR spectra of both complexes 2 and 3 showed characteristic multiplicity-free signals in the +55 to −25 ppm range (Fig. S10 and S11†), indicative of an open-shell electronic structure. Magnetic susceptibility measurements in C_6_D_6_ solution using Evans’ method^30^ yielded magnetic moments *μ*_eff_ = 4.87 and 4.88*μ*_B_ for complexes 2 and 3, respectively. These values are consistent with the spin-only value (4.90*μ*_B_) for a quintet (*S* = 2) spin system and high-spin iron(ii) complexes. These data are very similar to those of complex 1 and suggest a similar coordination environment around the Fe center. Furthermore, bulk purity of both compounds 2 and 3 was deduced from the corresponding CHN combustion elemental analysis.

**Fig. 1:**
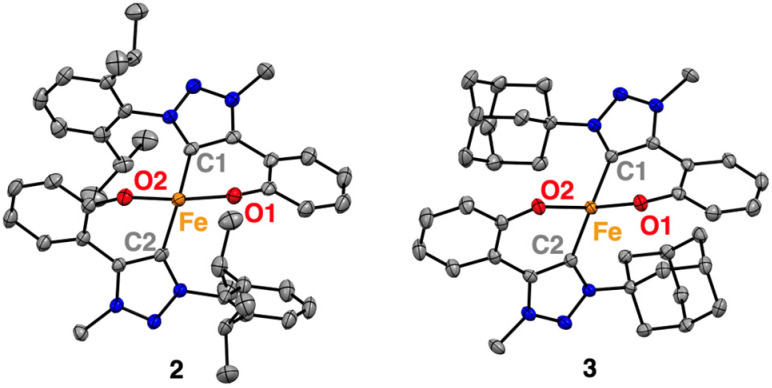
Molecular structures for complexes 2, and 3 determined by single-crystal X-ray diffraction analysis (50% probability ellipsoids, hydrogen atoms and any co-crystallised solvent molecules omitted for clarity).

Furthermore, single crystals of complexes 2 and 3 were grown from concentrated THF solutions upon slow diffusion of hexane. X-ray diffraction analysis confirmed the molecular structures deduced from solution analysis (Fig. 1). Both complexes feature homoleptic geometries, each possessing two C,O-bidentate chelating phenolate-carbene ligands coordinated to the metal centre. The complexes exhibit highly distorted tetrahedral structures with *τ*_4_ = 0.79–0.80, (*τ*′_4_ = 0.77; Table 1).^31,32^ The bite angle of the C,O-bidentate ligands is 90 ± 1° (Table 1), with the Fe–O bond lengths consistently at 1.945(5) Å, while the Fe–C bonds oscillate around 2.06(2) Å and vary slightly more across complexes 1–3. Notably, complex 2 and 3 feature the longest Fe(ii)–C_trz_ bonds known thus far for a monometallic complex with 2.0607(14) and 2.0827(14) Å, respectively.^33–37^ This bond lengthening may be a direct consequence of the steric bulk introduced at the wingtip substituents. These ligand modifications also induce a considerable widening of the O–Fe–O angle in complex 2 (128.69(4)°) similar to 1 (126.92(5)°) while in complex 3, this angle is 114.01(4)° and thus more commensurate with the 109.5° in an ideal tetrahedral coordination geometry. The larger angles for complexes 1 and 2 may be rationalized by electrostatic O⋯O repulsion together with interligand π-stacking of the aryl *N*-substituents with the phenolate of the other C,O-ligand (dipp⋯phenolate 3.603(1) Å‡).^38^ In contrast, the adamantyl substituents in complex 3 lack the potential for such interligand interactions. Instead, the enhanced 3-dimensional bulk increases the repulsion with the phenolate, which outweighs the electrostatic O⋯O repulsion of the two phenolate ligands.‡Stacking distance was calculated using Olex2-1.5 software by creating a plane between the phenolate ring connected to O2 and the dipp ring connected to C1 and using the ‘esd’ tool to calculate the distance between the centroids.

**Table 1:** Selected bond lengths (Å) and angles (°) for complexes 1–3^a^

	1^a^	2	3
Fe–C1	2.0407(12)	2.0554(14)	2.0827(14)
Fe–C2	2.0407(12)	2.0607(14)	2.0778(14)
Fe–O1	1.9474(9)	1.9402(10)	1.9518(10)
Fe–O2	1.9474(9)	1.9451(10)	1.9442(9)
O1–Fe–O2	126.91(5)	128.69(4)	114.01(4)
C1–Fe–O1	90.29(4)	89.96(5)	89.38(5)
C2–Fe–O2	90.29(4)	90.20(5)	89.50(5)
C1–Fe–O2	115.66(4)	114.66(5)	121.66(5)
C2–Fe–O1	115.66(4)	118.09(5)	126.92(5)
*τ*_4_ (*τ*′_4_)^b^	0.79 (0.78)	0.80 (0.77)	0.79 (0.77)

a Data for 1 from ref. 19, with C2 = C1′ and O2 = O1′.

b Calculated according to ref. 31 (*τ*_4_) and ref. 32 (*τ*′_4_).

### Catalytic C–H bond amination

**Fig. 2:**
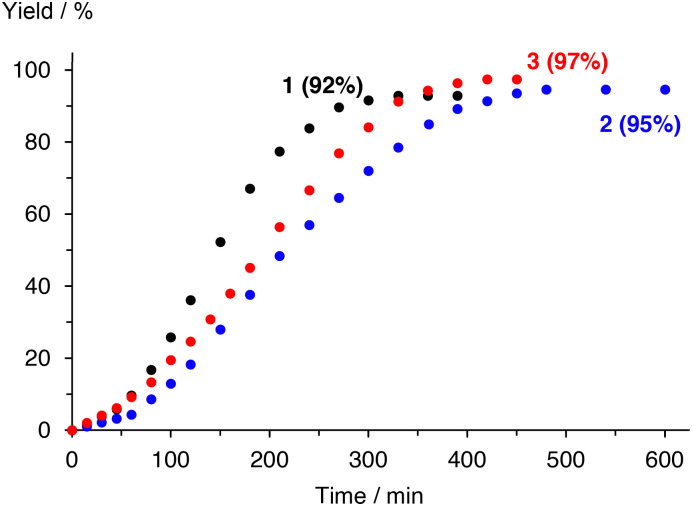
Time–yield profiles for the formation of C–H aminated product 5 with complexes 1–3. Conditions: [5]_0_ = 447.3 mM, and [Fe] = 4.47 mM (1 mol%), toluene-*d*_8_ (0.5 mL). Product quantification by ^1^H NMR spectroscopy using 1,3,5-trimethoxybenzene as internal standard.

Complexes 2 and 3 were evaluated as catalyst precursors for the intramolecular C–H amination utilizing 4-azido-4-methyl-pentyl-benzene 4 as an organic azide substrate to afford pyrrolidine product 5 (Table 2). Complexes 2 and 3 display activity very similar to that of complex 1 and reach almost quantitative conversion after 7 ± 1 h at 1 mol% catalyst loading and 120 °C. Comparison of the pertinent time–conversion profiles reveal subtle differences (Fig. 2). Complexes 2 and 3 reach their maximum turnover frequencies (TOF_max_) after *ca.* 3 h and thus later than complex 1 (1.5 h). Their TOF_max_ are slightly lower, 22 h^−1^ and 23 h^−1^ respectively, *vs.* 30 h^−1^ with complex 1, yet they all remain in the same order of magnitude (Table 2). With all complexes 1–3, the reaction proceeds to full conversion of the azide 4 according to ^1^H NMR spectroscopy, however the yield of pyrrolidine 5 is slightly higher for complex 2 than for 1 (95% *vs.* 92%) and almost quantitative for complex 3 (97%), indicating better suppression of side products such as amines and cyclic imines.

**Table 2:** Catalytic activity of complexes 1–3 in intramolecular C–H bond amination^a^

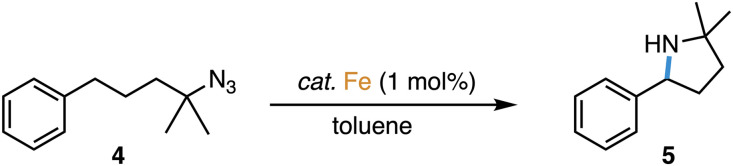				
Fe	Conversion 4	Yield 5	Time/h	TOF_max_/h^−1^
1	>99%	92%	6	30
2	>99%	95%	8	22
3	>99%	97%	7	23

a Reaction conditions: [4]_0_ = 447 mM and [Fe] = 4.47 mM (1 mol%) in toluene-*d*_8_ (0.5 mL) at 120 °C. TOF calculated from the time conversion profiles (Fig. 2).

The slightly decreased activity of complexes 2 and 3 suggests an energetically elevated transition state for the rate-limiting step. Mechanistic work with complex 1 has shown this step to be N_2_ loss from the coordinated azide to form the metal-coordinated nitrene intermediate.^19^ According to this model, N_2_ loss should be hampered by the increased steric requirements of the dipp and Ad groups in complexes 2 and 3 compared to 1. Speculatively, this difference may induce less strong azide coordination in these complexes and hence lower the catalytic activity, though obviously, also the longer Fe–C_trz_ bond may play a considerable role in increasing the Lewis acidity of the iron center. The same reasoning may rationalize the enhanced selectivity with complexes 2 and 3, as the increased steric protection limits the reactivity of the formed nitrene towards exogenous hydrogen atom sources, which would lead to terminal amine side products. Instead, the intramolecular insertion into the benzylic C–H bond is favored. According to this model, bonding of the pyrrolidine N-heterocyclic product, once formed, is sterically disfavored, thus preventing either hydrogen abstraction or dehydrogenation to form cyclic imine side products with these bulkier complexes 2 and especially 3.

**Fig. 3:**
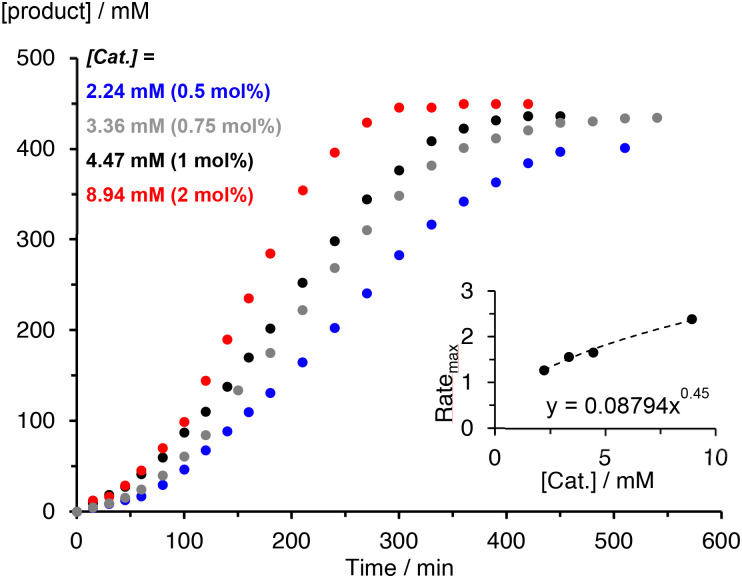
Kinetic profile of the C–H amination with complex 3 at varying concentrations of [3] = 2.24–8.94 mM, [4]_0_ = 447 mM, in toluene-*d*_8_. Product quantities determined by ^1^H NMR spectroscopy with 1,3,5-trimethoxybenzene as internal standard. Inset shows rate dependence on catalyst concentration and reveals 0.5 order rate dependence in complex (Rate_max_ in mM min^−1^; *R*^2^ = 0.993).

The kinetics of the C–H amination reaction catalyzed by complexes 2 and 3 were evaluated by varying the catalyst and initial substrate concentrations. Modulation of the loading of the iron complex 2 from 0.5, 0.75, 1, and 2 mol% provided quantitative conversions and product yields that remained at 95%. The time to reach full conversion increased to almost 14 h for the 0.5 mol% catalyst loading (Fig. S20†). For complex 3, the same variation had a moderate impact on the selectivity, with 90% yield at 0.5 mol% catalyst loading, 97% at 1 mol%, and >99% of pyrrolidine 5 at 2 mol% in just 6 h (Fig. 3). This essentially quantitative selectivity toward pyrrolidine formation indicates an efficient suppression of any side reactions such as undesired intermolecular hydrogen atom abstraction (Table 3).

**Table 3:** Dependence of catalyst performance on substrate and catalyst concentrations^a^

Entry	Fe	Conc./mM		Yield^b^	Time/h	TOF_max_/h^−1^
		[Fe]	[4]_0_			
1	2	4.47	447	95%	8	22
2	3	4.47	447	97%	7	23
3	2	2.24	447	96%	13.5	26
4	3	2.24	447	90%	8.5	38
5	2	8.94	447	95%	8	12
6	3	8.94	447	>99%	6	17
7	2	4.47	224	93%	12.5	7
8	3	4.47	224	92%	9.5	6
9	2	4.47	894	95%	7.5	45
10	3	4.47	894	>99%	7	51

a For general conditions, see Table 2.

b Yields determined by ^1^H NMR spectroscopy with 1,3,5-trimethoxybenzene as internal standard.

The time–yield profiles reveal for both complexes consistently a significant induction period of around 30–60 min (*cf.*Fig. 2). Moreover, the maximum rate of the reaction increases with increasing catalyst concentration, though the correlation is not linear and instead points to a half-order rate dependence in catalyst concentration for both complexes (inset Fig. 3 and S16†). This conclusion was also supported by the strong correlation of a variable time normalization analysis (VTNA)^39^ when assuming a 0.5 order in catalyst rather than a first-order dependence (Fig. S24–S28†). This dependence corroborates the mode of operation established for complex 1,^19^ and it therefore reinforces the significance of a dimeric species as catalyst resting state.

**Fig. 4:**
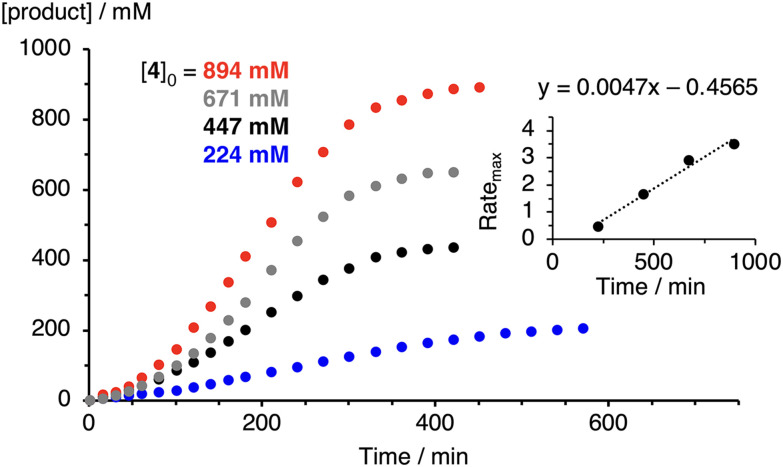
Kinetic profile of the C–H amination catalyzed by complex 3 at varying substrate concentrations. Conditions: [3] = 4.47 mM, [4]_0_ = 224–894 mM, toluene-*d*_8_. Product quantities determined by ^1^H NMR spectroscopy with 1,3,5-trimethoxybenzene as internal standard. Inset shows rate dependence on substrate concentration and reveals first order rate dependence in substrate (Rate_max_ in mM min^−1^, *R*^2^ = 0.997).

Similar variation of the substrate concentration gave full conversion at 1 : 50, 1 : 100, 1 : 150, and 1 : 200 complex/substrate ratios, albeit at longer reaction time for lower substrate concentrations (Table 3 and Fig. 4, S22, S23†). For example, at 1 : 50 ratio, >12 h were required to reach 93% yield with complex 2, while at a 1 : 200 catalyst/substrate ratio, 95% yield were accomplished in less than 8 h. Again, the reaction stoichiometry impacted the yield considerably more with complex 3, than the other two complexes. At low substrate concentration, the yield of pyrrolidine 5 leveled at only 92%, while at highest measured 0.9 M substrate concentration, full selectivity to the desired product was observed with >99% yield of 5. Notably, the changes of maximum rates correlate linearly with initial substrate concentrations, indicative of a first-order rate dependence with respect to substrate (Fig. 4 and Fig. S22, S23†).

The coherent appearance of an induction period, and the kinetic profile, especially the first-order dependence in substrate and the half-order in catalyst indicate that complexes 1–3 operate according to a common mechanism that is general for these types of iron carbene complexes. These data therefore reinforce the formation of a bimetallic catalyst resting state and a rate limiting step that involves this dimer and the substrate. While originally we proposed an azide- or nitrene-bridged dimer, one might also speculate about other dimeric species. For example, carbene–iron bond dissociation may be plausible when considering the high substitutional lability of iron(ii) paired with the scattered observations of carbene dissociation as catalyst activation pathways.^40–42^ Such a scenario would also be consistent with the observed induction time as the strongly bound chelating ligand is expected to require significant rearrangement for being cleaved from the iron center.^43^

## Conclusions

This work demonstrates that the catalytic C–H bond amination reactivity of iron triazolylidene complexes is directly affected by ligand modulations. Specifically, increasing bulk on the triazole *N*-substituent enhances the selectivity of the catalyst, accomplishing up to 99% yield of the pyrrolidine and thus suppressing efficiently side reactions that are typically observed in C–H amination such as the formation of terminal amines through nitrene quenching. Structural analysis of the complexes suggests that the smaller pocket available for substrate coordination reduces the propensity for intermolecular H atom abstraction, and thus maximizes the intramolecular reaction. At the same time, this smaller pocket also rationalizes the slightly lower activity when using bulkier ligands, as azide bonding is surmised to be weaker. These structure–activity trends indicate that the iron center remains ligated under catalytic conditions. In addition, the half-order rate dependence in iron complex is a common feature for this class of catalysts, suggesting a prominent role of dimers as catalyst resting states. These insights offer opportunities for further ligand modifications to rationally enhance catalytic activity.

## Experimental section

### General

Complex 1,^19^ 2-((trimethylsilyl)ethynyl)phenol,^44^ substrate 4,^1^ and organic azides dipp-N_3_,^45^ and Mes-N_3_ ^46^ were synthesised according to previously reported procedures. The synthesis of ligands L2 and L3 is described in the ESI.† All other reagents were commercially available and used as received unless stated otherwise. All manipulations involving the handling of transition metal complexes were performed in a MBraun glovebox with <0.1 ppm O_2_ and H_2_O levels using dry and degassed solvents. Benzene, hexane, pentane and diethyl ether were taken from an MBraun SPS system, degassed by three freeze–pump–thaw cycles and dried with 4 Å molecular sieves before use. THF was distilled from sodium benzophenone ketyl radical, degassed by three freeze–pump–thaw cycles and dried over 4 Å molecular sieves. THF-*d*_8_, C_6_D_6_ and toluene-*d*_8_ were distilled over NaK, degassed over three freeze–pump–thaw cycles and dried over 4 Å. Molecular sieves were pre-dried in a 1000 W microwave for 10 minutes, in 30 seconds intervals and subsequently dried under vacuum for 3 days. All organic syntheses were performed under aerobic conditions with commercially available solvents unless stated otherwise. All other chemicals were used as received without further purification from commercial sources. All ^1^H, ^13^C and ^19^F NMR spectra were recorded on a Bruker AVANCE III HD 300 or a Bruker AVANCE III HD 400 at room temperature. The chemical shifts are reported relative to SiMe_4_ using the chemical shift of residual solvent signals as reference. Mass spectrometric analyses were performed on a LTQ Orbitrap XL (Thermo Scientific) high resolution mass spectrometer, equipped with a static nano electrospray ion source using Econo12 platinated quartz emitters (New Objective Inc.). Determination of contents of carbon, hydrogen and nitrogen was performed on a Thermo Scientific Organic Elemental Analyzer. Air and moisture sensitive samples were sealed in Santis tin capsules for liquids (2.9 × 6 mm) inside an argon filled glovebox, taken outside the glovebox and measured directly.

### General synthetic procedure of complexes

In an argon-filled glovebox the solid ligand (2.0 eq.) was suspended in THF (2 mL) and cooled to −30 °C. A pre-cooled solution (−30 °C) of KHMDS (2.0 eq.) in THF (2 mL) was added dropwise to the suspension of ligand and stirred for 1 h at room temperature and subsequently cooled to −30 °C. A pre-cooled solution (−30 °C) of Fe(HMDS)_2_ (1.0 eq.) in THF (2 mL) was added dropwise to the ligand/KHMDS mixture and stirred for 18 h at room temperature. The reaction mixture was concentrated to approximately 1 mL and hexane (10 mL) was added. The resulting orange precipitate was collected by filtration, washed with Et_2_O (3 × 5 mL) and extracted into benzene. The resulting solution was freeze-dried and washed with pentane (5 × 5 mL) to obtain the title complexes.

### Complex 2

According to the general method, L2 (400 mg, 0.82 mmol), KHMDS (164 mg, 0.82 mmol) and Fe(HMDS)_2_ (155 mg, 0.41 mmol) afforded complex 2 as a bright orange powder (78 mg, 26%). Single crystals suitable for XRD analysis were obtained by laying a concentrated THF solution of the complex with hexane. ^1^H NMR (300 MHz, C_6_D_6_): *δ* = 47.7 (brs), 47.0 (brs), 18.0 (brs), 5.1 (brs), −1.9 (brs), −7.9 (brs), −22.6 (brs); *μ*_eff_ (C_6_D_6_, 299 K): 4.87*μ*_B_; elemental analysis calcd for C_42_H_48_FeN_6_O_2_ (724.73 g mol^−1^): C 69.61, H 6.68, N 11.60%. Found: C 69.97, H 6.81, N 11.21%.

### Complex 3

According to the general method, L3 (200 mg, 0.44 mmol), KHMDS (87 mg, 0.22 mmol) and Fe(HMDS)_2_ (82 mg, 0.22 mmol) yielded complex 3 as a bright orange powder (77 mg, 53%). Single crystals suitable for XRD analysis were obtained by laying a concentrated THF solution of 3 with hexane. ^1^H NMR (300 MHz, THF-*d*_8_): *δ* = 54.2 (brs), 51.0 (brs), 18.4 (brs), −0.1 to −1.8 (m), −14.7 (brs); *μ*_eff_ (C_6_D_6_, 299 K): 4.88*μ*_B_; elemental analysis calcd for C_38_H_44_FeN_6_O_2_ (672.66 g mol^−1^): C 67.85, H 6.59, N 12.44%. Found: C 67.59, H 6.77, N 12.53%.

### General catalytic procedure

Inside an argon filled glovebox, a stock solution of complex was prepared by dissolving a known amount of iron complex in toluene-*d*_8_ (2 mL). A stock solution of standard was prepared by dissolving 1,3,5-trimethoxybenzene (45.5 mg. 0.0271 mmol) in toluene-*d*_8_ (1.0 mL). The azide 4 (25, 50, 75, or 100 mg) was weighed into a vial, portions of the iron complex stock solution (0.2 mL), internal standard solution (0.1 mL), and toluene-*d*_8_ (0.2 mL) were added. This mixture was transferred into an oven-dried J Young NMR tube, transported out of the glove box, and heated in an oil bath at 120 °C. Conversion was measured at given time intervals after removing the NMR tube from the oil bath and cooling it in an ice bath before measuring a ^1^H NMR spectrum. Times listed are the cumulative amount of time in the oil bath at the specified temperature. Yields were determined by ^1^H NMR analysis relative to the internal standard following the proton signal of the benzylic hydrogens of 4 at 2.28 ppm (toluene-*d*_8_) for substrate and the appearance of a quartet at 4.03 ppm (toluene-*d*_8_) corresponding to the cyclized product 5 (Fig. S18 and S19†). Lower catalyst loadings were achieved by appropriate dilution of the complex stock solution to maintain a constant reaction volume.

## Data availability

The data supporting this article have been included as part of the ESI.† Crystallographic data for complexes 2 and 3 have been deposited at the CCDC under 2361237 and 2361238.†

## Conflicts of interest

The authors declare no competing financial interest.

## Supplementary Material

DT-053-D4DT01715C-s001

DT-053-D4DT01715C-s002
