# Supplementary material for: Tailoring C–H amination activity via modification of the triazole-derived carbene ligand
Source: Dalton Trans. 2024 Aug 15;53(35):14795–800. doi: 10.1039/d4dt01715c (PMC11334762; doi:10.1039/d4dt01715c)
Supplement: DT-053-D4DT01715C-s001 [file DT-053-D4DT01715C-s001.pdf]

Supplementary Information (SI) for Dalton Transactions.
This journal is © The Royal Society of Chemistry 2024

Electronic Supporting Information

Tailoring C–H amination activity via modification of the triazole-derived carbene ligand

Luke A. Hudson, Wowa Stroek, and Martin Albrecht*

Department of Chemistry, Biochemistry and Pharmaceutical Sciences, University of Bern, Freiestrasse 3, 3012 Bern (Switzerland)

Email: martin.albrecht@unibe.ch

Table of Contents

1. Synthesis of ligand precursors L2 and L3 .... S2
2. Analytical Data for Complexes 2 and 3 .... S11
3. Catalytic Details S16
4. Crystallographic Details S27
5. References S29

1. Synthesis of ligand precursors L2 and L3

Synthesis of 2-(1-(2,6-diisopropylphenyl)-1H-1,2,3-triazol-4-yl)phenol

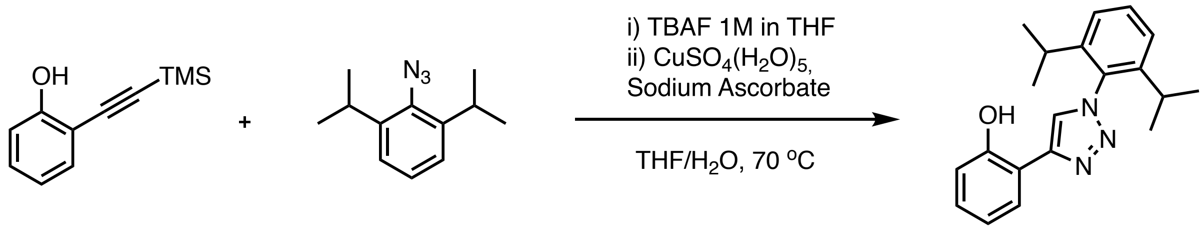

2-((trimethylsilyl)ethynyl)phenol (2.34 g, 12.30 mmol, 1.0 eq.) and 2-azido-1,3-diisopropylbenzene (2.50 g, 12.30 mmol, 1.0 eq.) were dissolved in THF (20 mL) and Bu_4NF 1M as a THF solution (13.53 mL, 13.53 mmol, 1.1 eq.) was added dropwise. After stirring for 2 h, $CuSO_4(H_2O)_5$ (614 mg, 2.46 mmol, 0.2 eq.) and sodium ascorbate (2.44 g, 12.30 mmol, 1.0 eq.) were added as a H_2O solution (20 mL) . The resulting mixture was then stirred at 70 °C for 40 h. Then the reaction mixture was concentrated *in vacuo* to yield a brown solid which was washed with H_2O (50 mL) and pentane (50 mL). The brown solid was redissolved in CH_2Cl_2 and washed with an aqueous NH_4OH solution (10%, 5 x 25 mL), H_2O (25 mL) and brine (25 mL). The resulting organic residue was dried over $MgSO_4$, filtered, and concentrated under reduced pressure to yield a pale brown powder (2.54 g, 64%)

^{1}H NMR (300 MHz, CD_2Cl_2) δ 10.87 (s, 1H, OH), 8.03 (s, 1H, H_{trz}), 7.62 – 7.53 (m, 2 x H, H_{Ph}, H_{dipp}), 7.40 – 7.34 (m, 2 x H, $H_{Ar,}$ H_{dipp}), 7.29 (t, J = 1.6 Hz, 1H, H_{Ar}), 7.11 – 7.05 (m, 1H, H_{Ar}), 6.96 (t, J = 1.2 Hz, 1H, H_{Ar}), 2.29 (heptet, J = 7.0 Hz, 2H, H_{iPr}), 1.17 (d, J = 6.9 Hz, 12H, $H_{iPr}CH_3$). **^{13}C{^{1}H} NMR** (75 MHz, CD_2Cl_2) δ 156.4 (C_{Ar}–OH), 147.8 (C_{Ar}–trz), 146.5 (2C, C_{dipp}–^{i}Pr) , 133.2 (C_{dipp}–trz), 131.6 (C_{dipp}–H), 130.3, 126.6 (2 x C, C_{Ar}–H), 124.4 (2C, C_{dipp}–H), 122.6 (C_{trz}–H), 120.0 (C_{Ar}–H), 118.0 (C_{Ar}–H), 114.3 (C_{trz}) , 28.9 (2C, C_{iPr}–$(CH_3)_2$, 24.5, 24.1 (2 x C, $C_{iPr}CH_3$). **HR-MS** Calc. for $C_{20}H_{24}ON_3$ [M + H], 322.1914. Found 322.1922

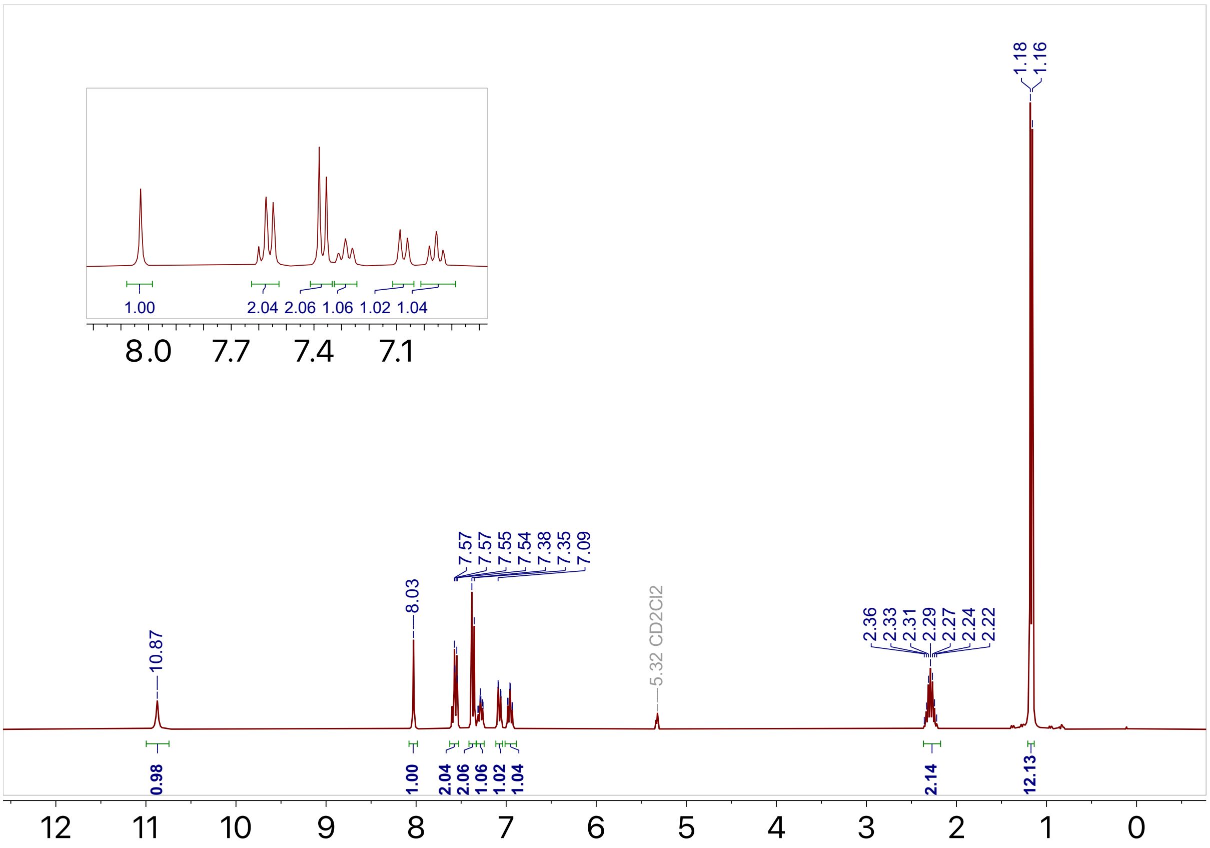

Figure S1: ^{1}H NMR spectrum (CD_2Cl_2, 298 K, 300 MHz) of 2-(1-(2,6-diisopropylphenyl)-1H-1,2,3-triazol-4-yl)phenol.

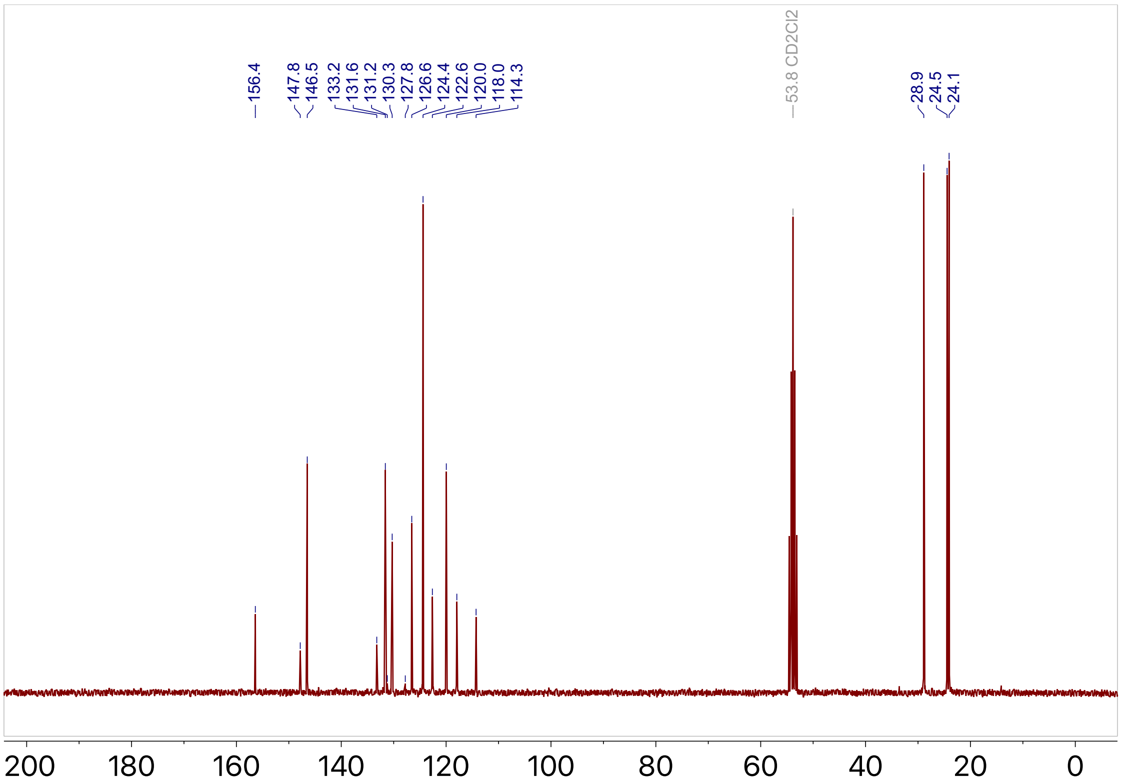

Figure S2: $^{13}C\{^{1}H\}$ NMR spectrum (CD_2Cl_2, 298 K, 75 MHz) of 2-(1-(2,6-diisopropylphenyl)-1H-1,2,3-triazol-4-yl)phenol.

Synthesis of L2

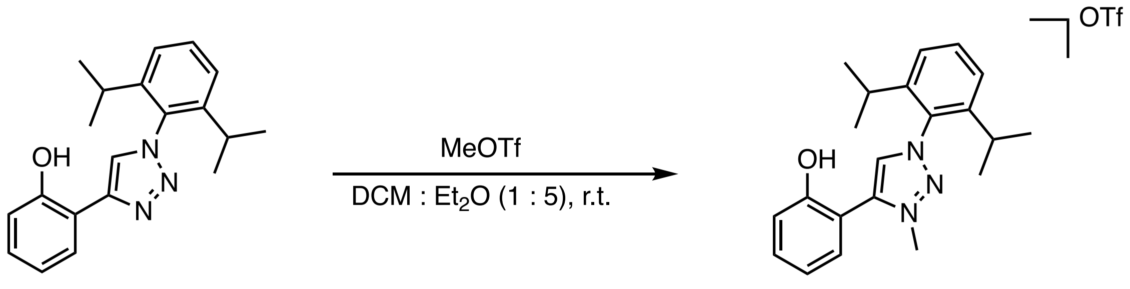

L2

2-(1-(2,6-diisopropylphenyl)-1H-1,2,3-triazol-4-yl)phenol (1.00g, 1.24 mmol, 1.0 eq.), was dissolved in a CH_2Cl_2/Et_2O (30 mL, 1:5 v/v) mixture and MeOTf (0.68 mL, 2.48 mmol, 2.0 eq.) was added dropwise. The resulting mixture was stirred at room temperature for 6 h over which time a precipitate was observed to have formed. The precipitate was collected by filtration to give a pale pink powder (1.03 g, 68%).

^{1}H NMR (300 MHz, CD_2Cl_2) δ 8.36 (s, 1H, H_{trz}), 7.69 (t, J = 7.8 Hz, 1H, H_{dipp}), 7.53 (t, J = 7.8 Hz, 1H, H_{Ar}), 7.48 – 7.34 (m, 4H, 2 x H_{dipp}, 2 x H_{Ar}), 7.10 (t, J = 7.5 Hz, 1H, H_{Ar}), 4.42 (s, 3H, $H_{trz}CH_3$), 2.27 (heptet, J = 6.9 Hz, 2H, H_{iPr}), 1.23 (t, J = 6.2 Hz, 12H, $H_{iPr}CH_3$). **^{13}C{^{1}H} NMR** (75 MHz, CD_2Cl_2) δ 156.4 (C_{Ar}–OH), 145.8 (2C, C_{dipp}–iPr), 142.8 (C_{Ar}–trz), 134.9 (C_{Ar}–H), 133.5 (C_{dipp}–H), 131.7 (C_{trz}–H), 131.0 (C_{dipp}–trz), 130.8 (C_{Ar}–H), 125.3 (2C, C_{dipp}–H) ,120.8 (C_{Ar}–H), 118.2 (C_{Ar}–H), 108.3 (C_{trz}), 40.3 (C_{trz}–CH_3), 29.3 (2C, C_{iPr}–$(CH_3)_2$), 24.4, 23.9 (2 x C, $C_{iPr}CH_3$). **^{19}F NMR** (282 MHz, CD_2Cl_2) δ -79.03. **HR-MS** Calc. for $C_{21}H_{26}ON_3$ [M – OTf^-], 336.2070. Found 336.2065. **Elemental Analysis** Calc. for $C_{25}H_{34}F_3N_3O_4S$ (485.52 g mol^{-1}): C 54.42, H 5.40, N 8.65. Found C 54.30, H 5.32, N 8.65.

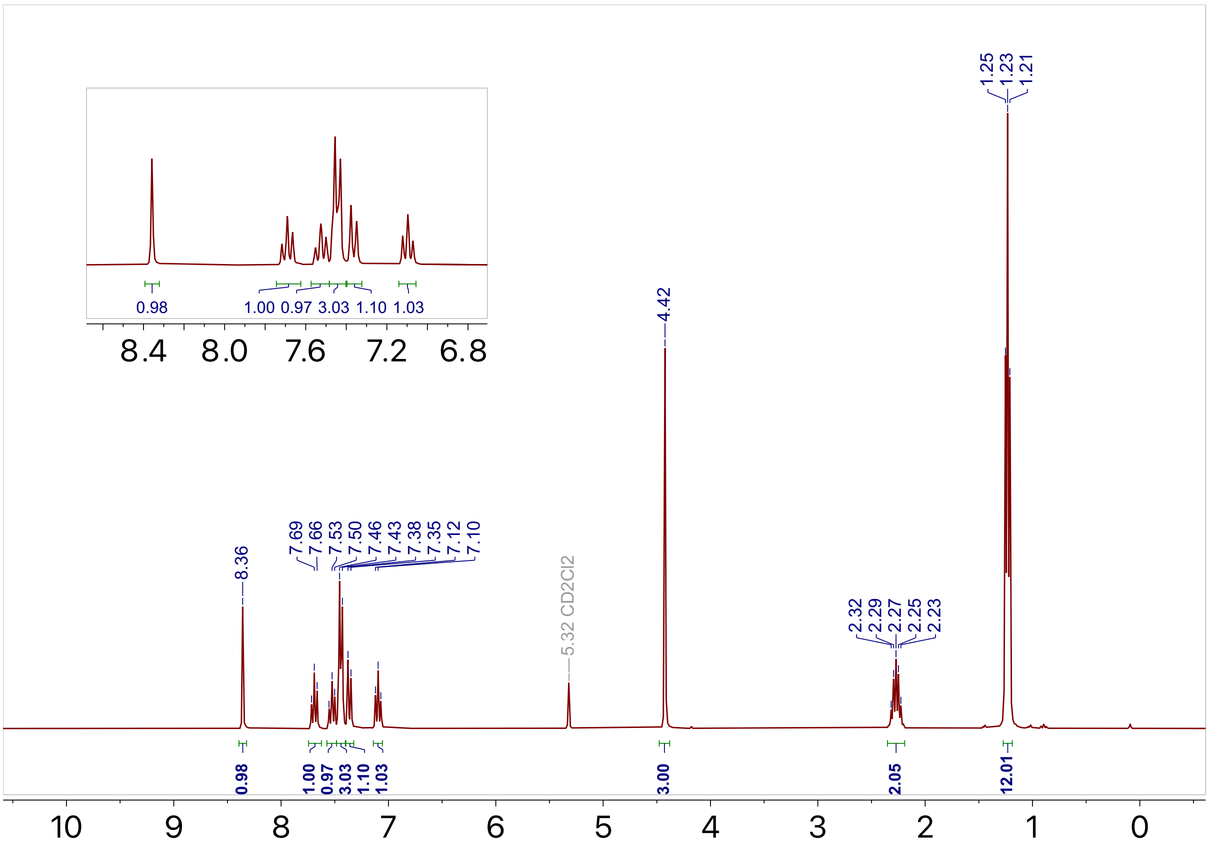

Figure S3: ^{1}H NMR spectrum (CD_2Cl_2, 298 K, 300 MHz) of **L2**.

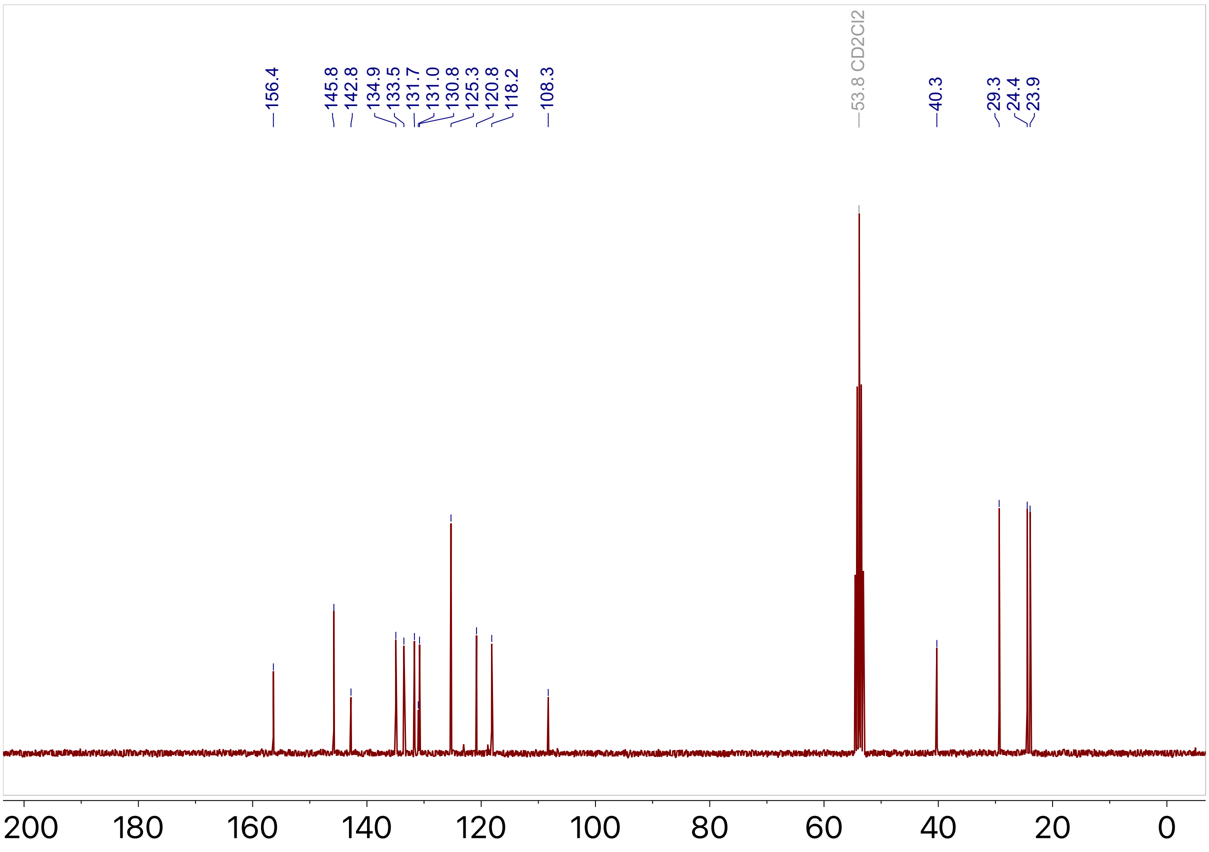

Figure S4: $^{13}C\{^{1}H\}$ NMR spectrum (CD_2Cl_2, 298 K, 75 MHz) of **L2**.

Synthesis of 2-(1-((3s,5s,7s)-adamantan-1-yl)-1H-1,2,3-triazol-4-yl)phenol

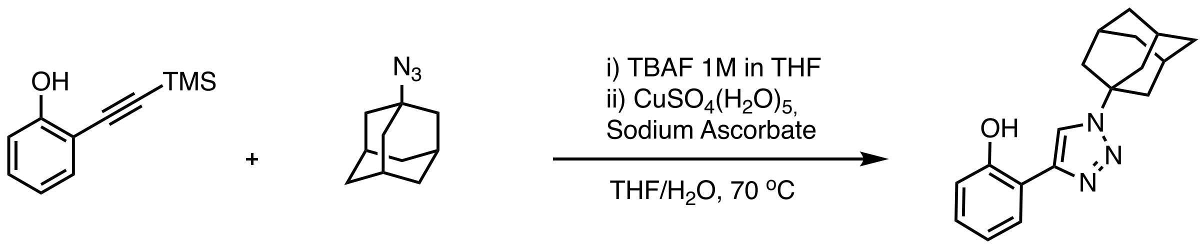

2-((trimethylsilyl)ethynyl)phenol (1.61 g, 8.46 mmol, 1.0 eq.) and 1-azidoadamantane (1.50 g, 8.46 mmol, 1.0 eq.) were dissolved in THF (10 mL) and Bu_4NF 1M as a THF solution (9.31 mL, 9.31 mmol, 1.1 eq.) was added dropwise. After stirring for 2h, $CuSO_4(H_2O)_5$ (423 mg, 1.69 mmol, 0.2 eq.) and sodium ascorbate (1.68 g, 8.46 mmol, 1.0 eq.) were added as a H_2O solution (20 mL). The resulting mixture was stirred at 70°C for 40 h. The reaction mixture was then concentrated *in vacuo*, extracted with CH_2Cl_2 (3 x 30 mL) and then washed with NH_4OH solution (10%, 5 x 30 mL) and brine (30 mL). The resulting organic residue was dried over Na_2SO_4, filtered and concentrated under reduced pressure to yield a brown oil. The brown oil was then added dropwise to pentane to yield an off-white solid that was collected by filtration (**454mg, 18%**)

^{1}H NMR (300 MHz, CD_2Cl_2) δ 7.97 (s, 1H, H_{trz}), 7.48 (d, J = 7.7 Hz, 1H, H_{Ar}), 7.22 (t, J = 7.8 Hz, 1H, H_{Ar}), 7.11 – 6.75 (m, 2H, H_{Ar}), 2.47 – 2.13 (m, 9H, H_{Ad}), 2.00 – 1.72 (m, 6H, H_{Ad}).**^{13}C{^{1}H} NMR** (75 MHz, CD_2Cl_2) δ 156.3 (C_{Ar}–OH), 147.0 (C_{Ar}–trz), 129.7 (C_{Ar}–H), 126.1 (C_{Ar}–H), 119.8 (C_{Ar}–H), 117.7 (C_{Ar}–H), 116.1 (C_{trz}), 114.8 (C_{trz}–Ar), 43.4 (C_{Ad}), 36.2 (C_{Ad}), 30.1 (C_{Ad}). **HR-MS** Calc. for $C_{18}H_{22}N_3O$ [M + H], 296.1757. Found 296.1764.

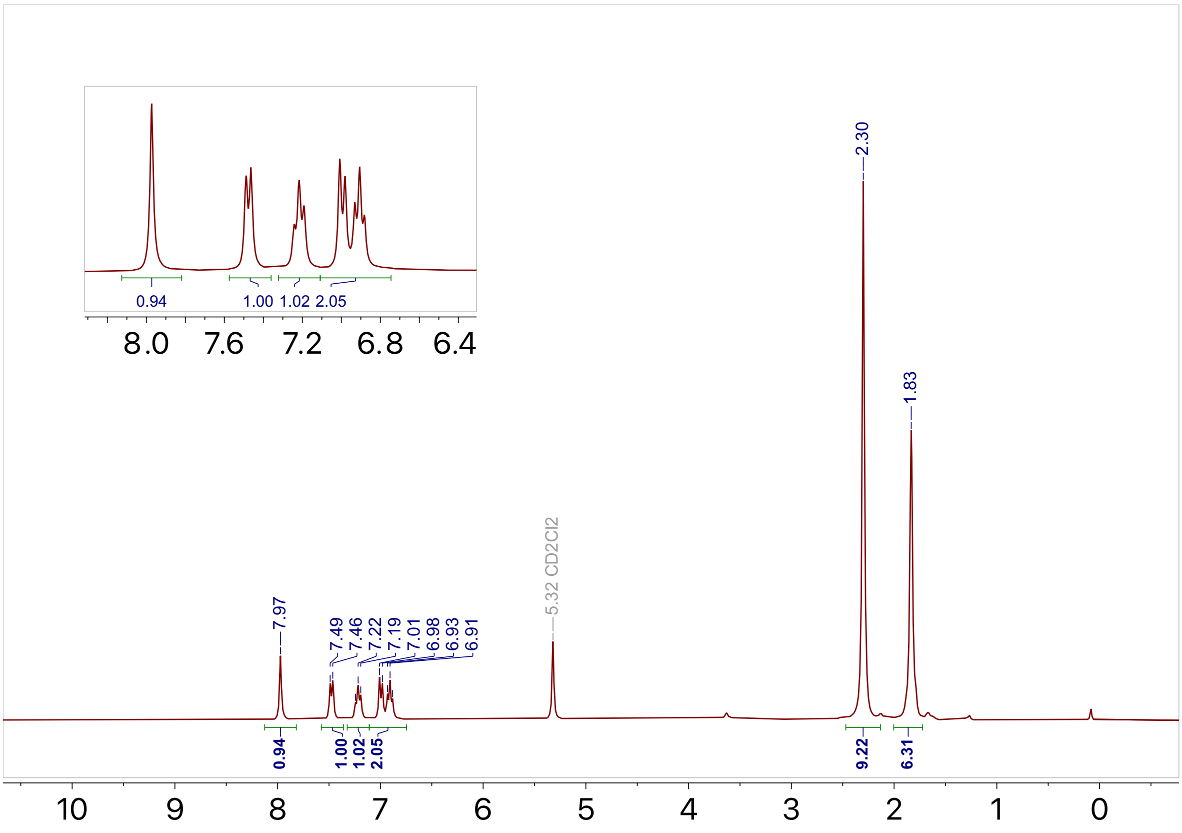

Figure S5: ^{1}H NMR spectrum (CD_2Cl_2, 298 K, 300 MHz) of 2-(1-((3s,5s,7s)-adamantan-1-yl)-1H-1,2,3-triazol-4-yl)phenol..

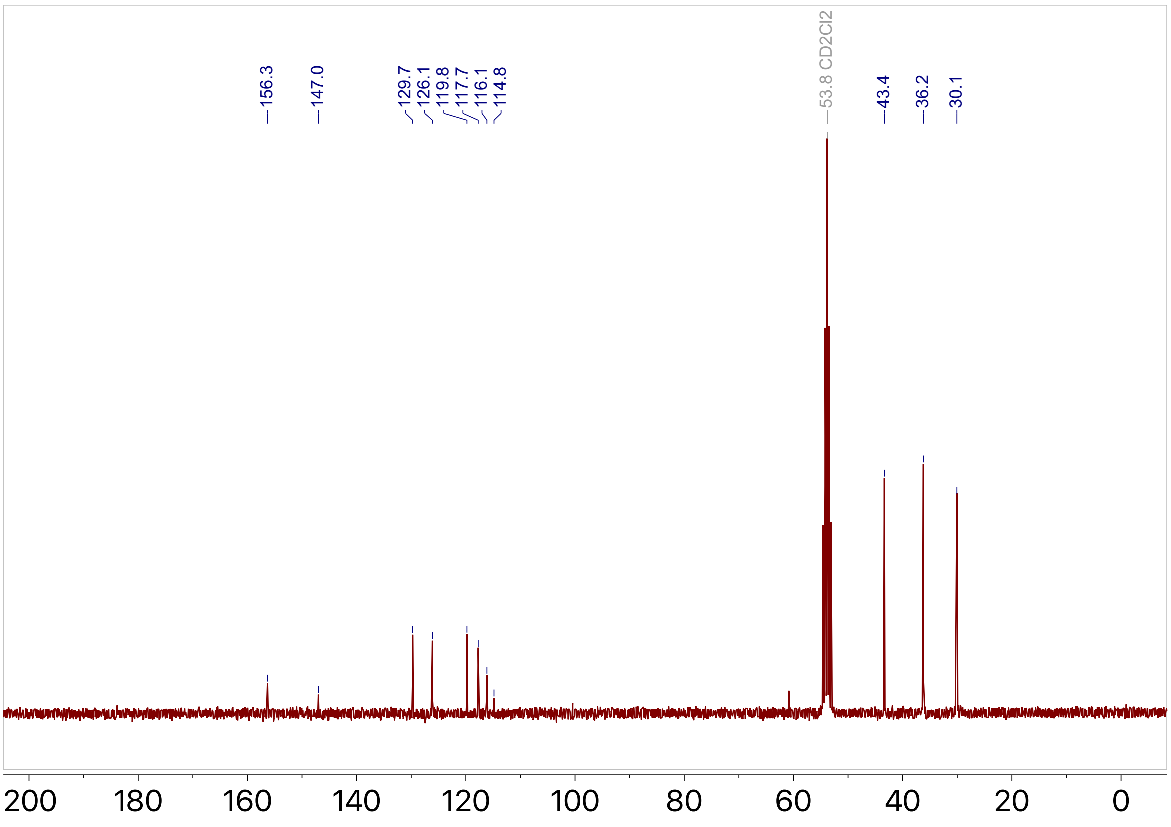

Figure S6: $^{13}C\{^{1}H\}$ NMR spectrum (CD_2Cl_2, 298 K, 75 MHz) of 2-(1-((3s,5s,7s)-adamantan-1-yl)-1H-1,2,3-triazol-4-yl)phenol.

Synthesis of L3

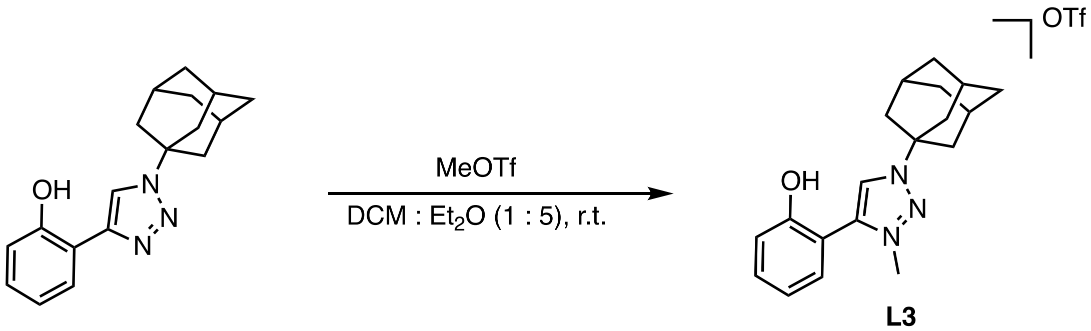

L3

2-(1-((3s,5s,7s)-adamantan-1-yl)-1H-1,2,3-triazol-4-yl)phenol (350 mg, 1.18 mmol, 1.0 eq.) was dissolved in a CH_2Cl_2:Et_2O (12 mL, 1:5 v/v) mixture and MeOTf (0.26 mL, 2.36mmol, 2.0 eq.) was added dropwise to give a dark brown solution which was stirred for 6 h at room temperature over which time a precipitate formed which was collected by filtration to yield an off-white powder (**407 mg, 75%**)

^{1}H NMR: (300 MHz, CD_2Cl_2) δ 9.35 (s, 1H, OH), 8.27 (s, 1H, H_{trz}), 7.48 (ddd, J = 8.9, 7.4, 1.7 Hz, 1H, H_{Ar}), 7.36–7.20 (m, 2H, H_{Ar}), 7.03 (ddd, J = 7.5, 7.4, 1.1 Hz, 1H, H_{Ar}), 4.21 (s, 3H, $H_{trz}CH_3$), 2.41–2.29 (m, 9H, H_{Ad}), 1.92–1.75 (m, 6H, H_{Ad}).**^{13}C{^{1}H} NMR:** (75 MHz, CD_2Cl_2) δ 156.1 (C_{Ar}–OH), 142.0 (C_{Ar}–trz), 134.5 (C_{Ar}–H), 130.9 (C_{Ar}–H), 125.3 (C_{trz}–H), 120.8 (C_{Ar}–H), 118.2 (C_{Ar}–H), 109.1 (C_{trz}–Ar), 66.9 (C_{Ad}–trz), 42.5 (C_{Ad}CH), 39.5 ($C_{trz}CH_3$), 35.6 (C_{Ad}CH), 30.0 ($C_{Ad}CH_2$). **^{19}F NMR:** (282 MHz, CD_2Cl_2) δ -78.99. **HR-MS:** Calc. for $C_{19}H_{24}ON_3$ [M – OTf^-], 310.1914. Found 310.1907. **Elemental Analysis:** Calc. for $C_{20}H_{24}F_3N_3O_4S$ (459.48 g mol^{-1}): C 52.28, H 5.27, N 9.19. Found C 52.22, H 5.05 N 8.91.

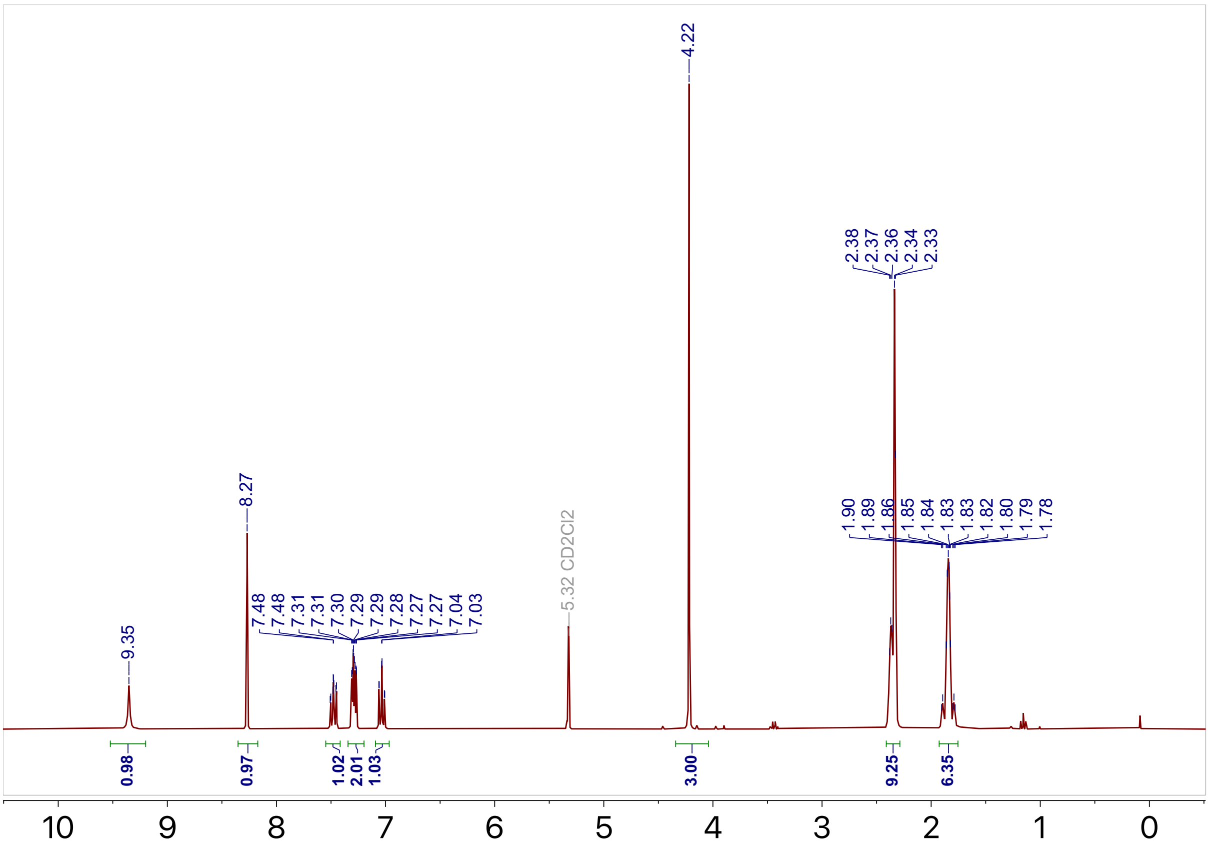

Figure S7: ^{1}H NMR spectrum (CD_2Cl_2, 298 K, 300 MHz) of compound **L3**.

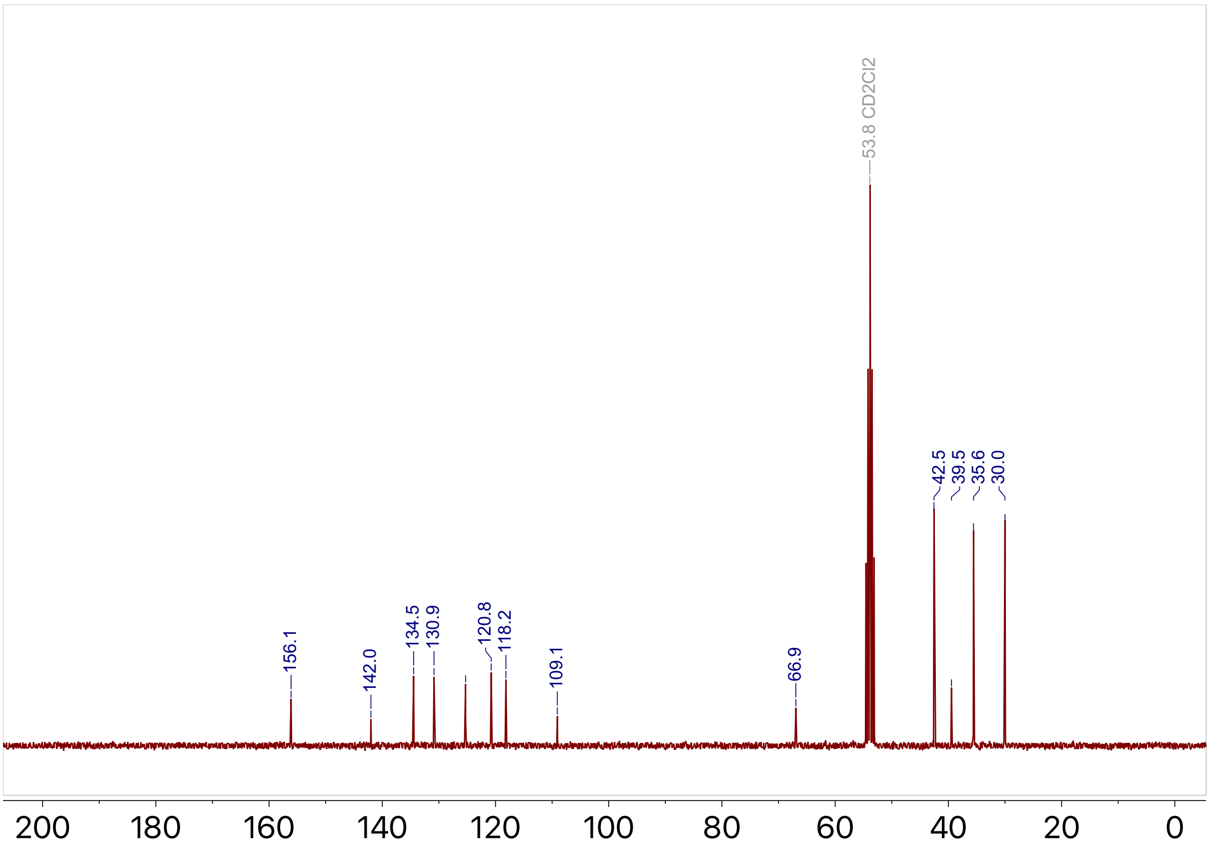

Figure S8: $^{13}C\{^{1}H\}$ spectrum (CD_2Cl_2, 298 K, 75 MHz) of compound **L3**.

Thermal Elemental Analysis

Name: **Luke Hudson**

Summarize Results
Date : 7/7/2023 8:32:17
Method Name : CHN_fluorinated_Compounds
Method Filename : CHN_F 2023_07_06.mth

Sample name	Anal. Date	Inj. Time	(mg)	% Nitrogen	% Carbon	% Hydrogen
**LAH 068 a**	7/6/2023	13:02	1.326	8.96	52.00	5.10
**LAH 068 b**	7/6/2023	13:10	1.116	8.95	52.18	5.09
**LAH 068 c**	7/6/2023	13:18	0.552	8.82	52.49	4.97
3 Sample(s) in Group No : 8						
Component Name	Average	Std. Dev.	% Rel. S. D.		theor.	dev.
**Nitrogen**	**8.91**	**0.078**	**0.877**		**9.19**	**-0.28**
**Carbon**	**52.22**	**0.248**	**0.475**		**52.28**	**-0.06**
**Hydrogen**	**5.05**	**0.072**	**1.432**		**5.27**	**-0.22**

Sample name	Anal. Date	Inj. Time	(mg)	Nitrogen	Carbon	Hydrogen
**LAH 066 a**	7/6/2023	13:27	1.688	8.69	54.25	5.35
**LAH 066 b**	7/6/2023	13:35	1.274	8.62	54.43	5.25
**LAH 066 c**	7/6/2023	13:43	1.538	8.64	54.21	5.35
3 Sample(s) in Group No : 9						
Component Name	Average	Std. Dev.	% Rel. S. D.		theor.	dev.
**Nitrogen**	**8.65**	**0.036**	**0.417**		**8.65**	**0.00**
**Carbon**	**54.3**	**0.117**	**0.216**		**54.42**	**-0.12**
**Hydrogen**	**5.32**	**0.058**	**1.086**		**5.40**	**-0.08**

Figure S9: CHN combustion analysis report of ligand precursors **L2** (LAH066) and **L3** (LAH068).

2. Analytical Data for Complexes 2 and 3

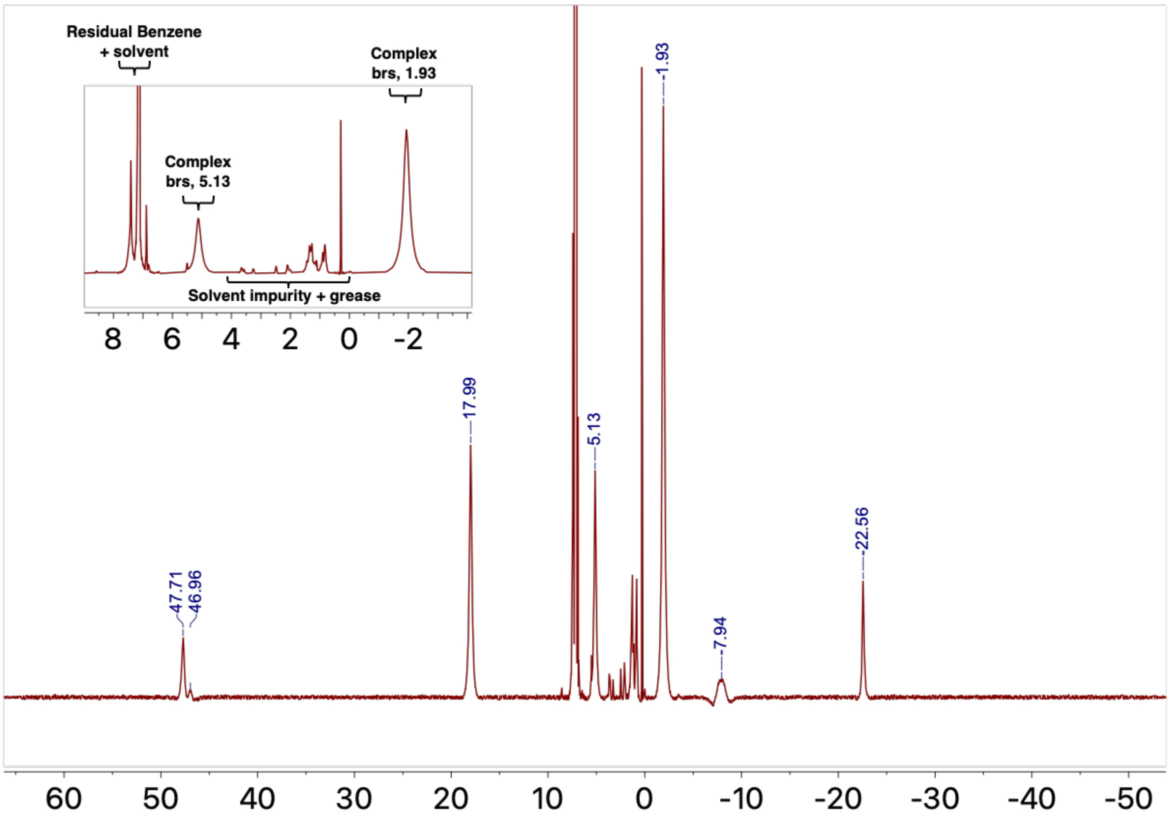

Figure S10: ^{1}H NMR spectrum (C_6D_6, 298 K, 300 MHz) of complex **2**.

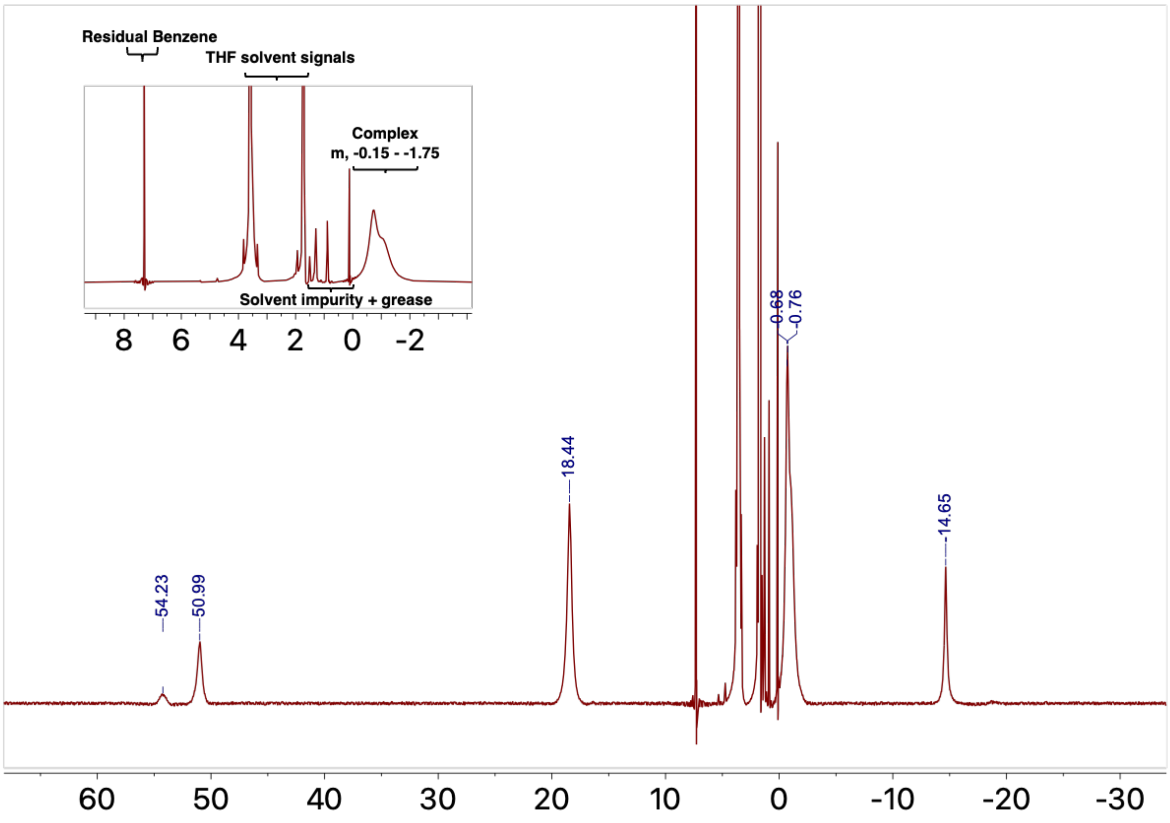

Figure S11: ^{1}H NMR spectrum (THF-d_8, 298K, 300 MHz) of complex **3**.

Thermal Elemental Analysis

Name: **Luke Hudson**

Summarize Results
Date : 13.10.2023 09:38:15
Method Name : CHN_fluorinated_Compounds
Method Filename : CHN_F 2023_10_12.mth

Sample name	Anal. Date	Inj. Time	(mg)	Nitrogen	Carbon	Hydrogen
**LAH141_14**	12.10.2023	11:40	1.346	11.18	69.80	6.72
**LAH141_10**	12.10.2023	11:49	1.356	11.22	70.11	6.85
**LAH141_07**	12.10.2023	11:57	1.424	11.24	70.00	6.85
3 Sample(s) in Group No : 4						
Component Name	Average	Std. Dev.	% Rel. S. D.		theor.	dev.
**Nitrogen**	**11.21**	**0.031**	**0.272**		**11.60**	**-0.39**
**Carbon**	**69.97**	**0.157**	**0.225**		**69.61**	**0.36**
**Hydrogen**	**6.81**	**0.075**	**1.103**		**6.68**	**0.13**

Figure S12: Elemental analysis of complex **2.**

Thermal Elemental Analysis

Name: **Luke Hudson**

Summarize Results
Date : 13.10.2023 09:38:15
Method Name : CHN_fluorinated_Compounds
Method Filename : CHN_F 2023_10_12.mth

Sample name	Anal. Date	Inj. Time	(mg)	% Nitrogen	% Carbon	% Hydrogen
**LAH142_12**	12.10.2023	11:31	1.808	12.53	67.59	6.77
1 Sample(s) in Group No : 3						
Component Name	Average	Std. Dev.	% Rel. S. D.		theor.	dev.
**Nitrogen**	**12.53**				**12.44**	**0.09**
**Carbon**	**67.59**				**67.85**	**-0.26**
**Hydrogen**	**6.77**				**6.59**	**0.18**

Figure S13: Elemental analysis report of Complex **3.**

Magnetic susceptibility measurements for complexes 2 and 3

Magnetic data in solution was measured using Evans Method.[S1] A known amount of analyte was fully dissolved in a known amount of C_6D_6 with 20% non-deuterated toluene as internal standard and transferrd into an NMR tube. A capillary was filled with the same solution without analyte and inserted into the NMR tube with the analyte solution. A ^{1}H NMR spectrum was recorded at a known temperature and Δv was determined from the chemical shifts of the internal standard (Hz). The molar susceptibility (χ_m) was calculated according to equation S1, in which v_0 is the frequency of the NMR spectrometer (Hz) and *c* as the concentration of the analyte (M). The diamagnetic molar susceptibility (χ_m^{Dia}) was calculated according ot equation S2, in which MW is the molecular weight. The paramagnetic molar susceptibility (χ_m^{Para}) was calculated according to equation S3. Finally, the effective magnetic moment (μ_{eff}) was calculated according to equation S4, in which *T* is the temperature in K during the measurement.

$$\chi_m = \frac{3000 \cdot \Delta\nu}{4\pi \cdot \nu_0 \cdot c} \qquad (Equation\ S1)$$

$$\chi_m^{Dia} = -\frac{MW}{2 \cdot 10^6} \qquad (Equation\ S2)$$

$$\chi_m^{Para} = \chi_m - \chi_m^{Dia} \qquad (Equation\ S3)$$

$$\mu_{eff} = 2.82787\sqrt{\chi_m^{Para} \cdot T} \qquad (Equation\ S4)$$

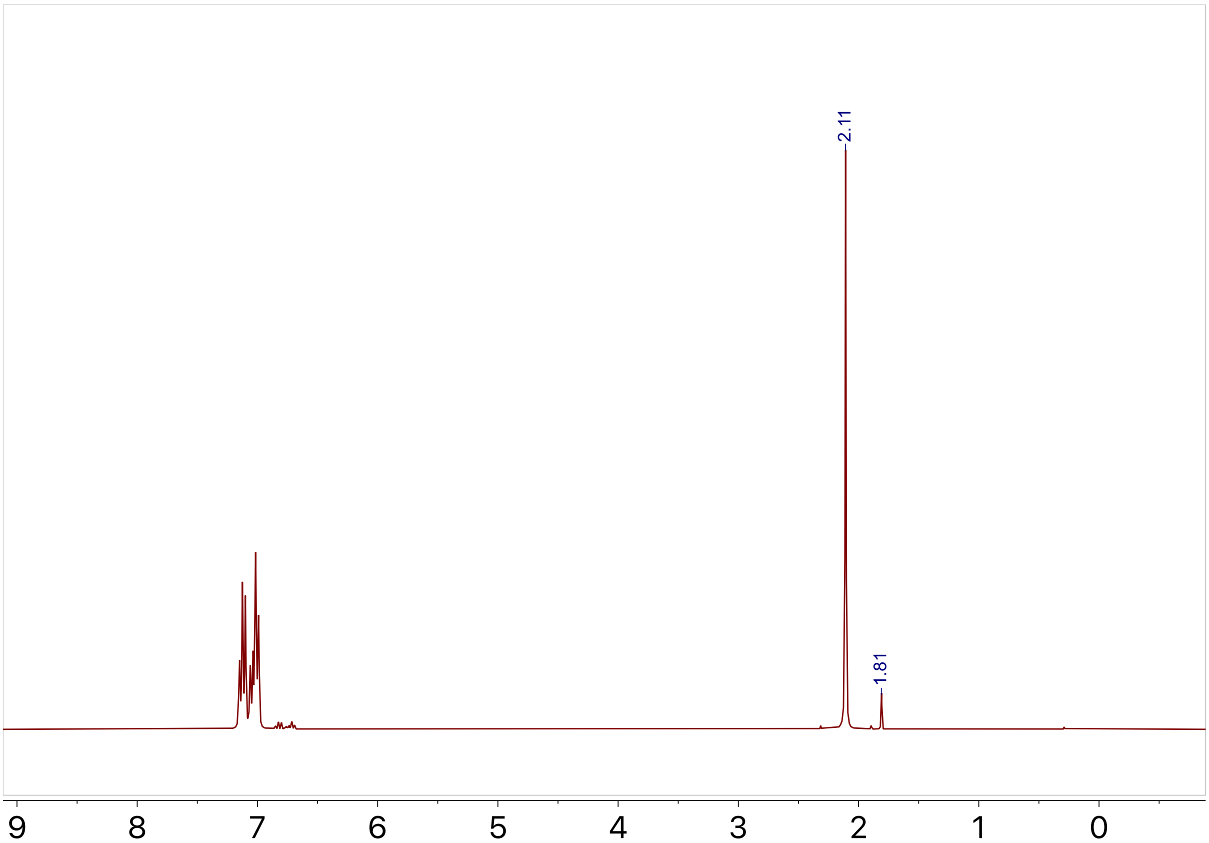

Figure S14: Evans method of 7.5 mM solution of complex **2** in C_6D_6 and toluene as internal standard at 299 K.

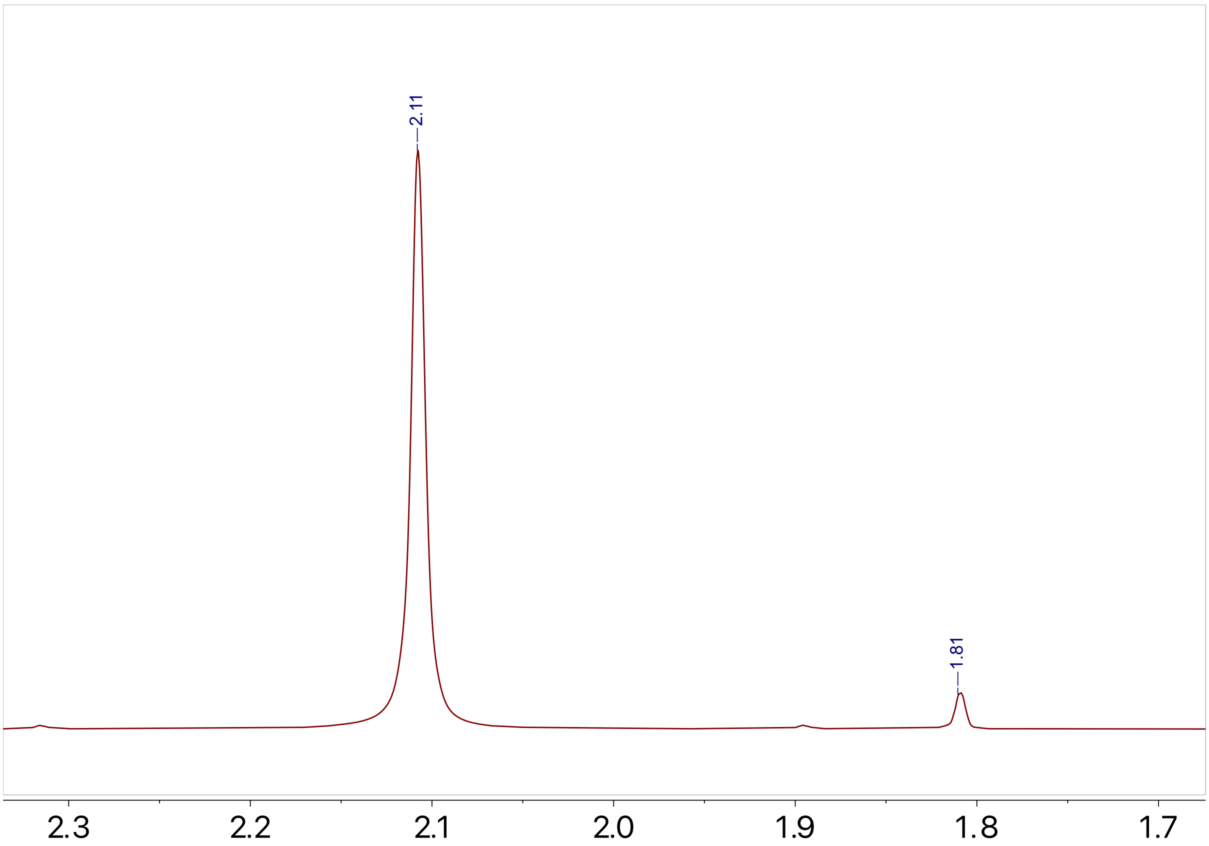

Figure S15: Evans method of 7.5 mM solution of complex **2** in C_6D_6 with toluene as internal standard at 299 K, zoomed in on the CH_3 signal of the toluene (internal standard)

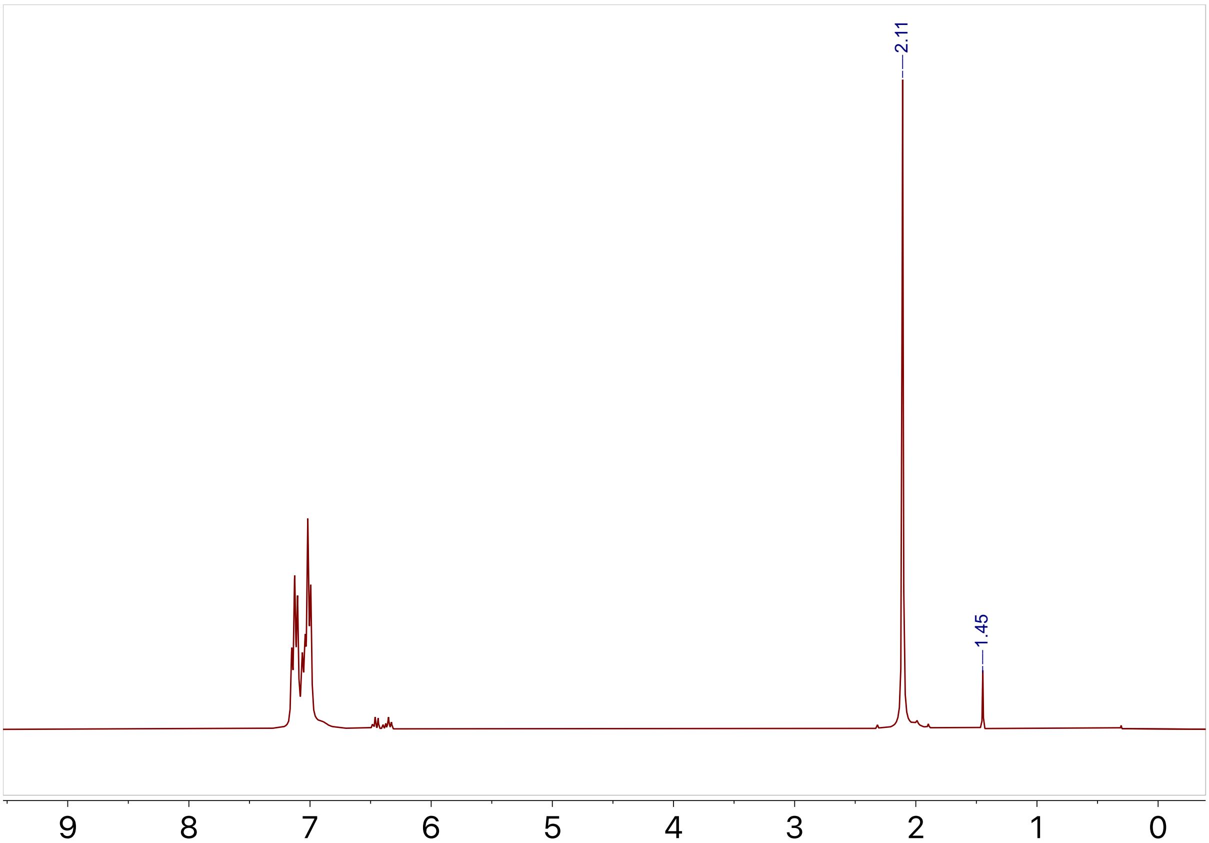

Figure S16: Evans method of 16.4 mM solution of complex **3** in C_6D_6 and toluene as internal standard at 299 K.

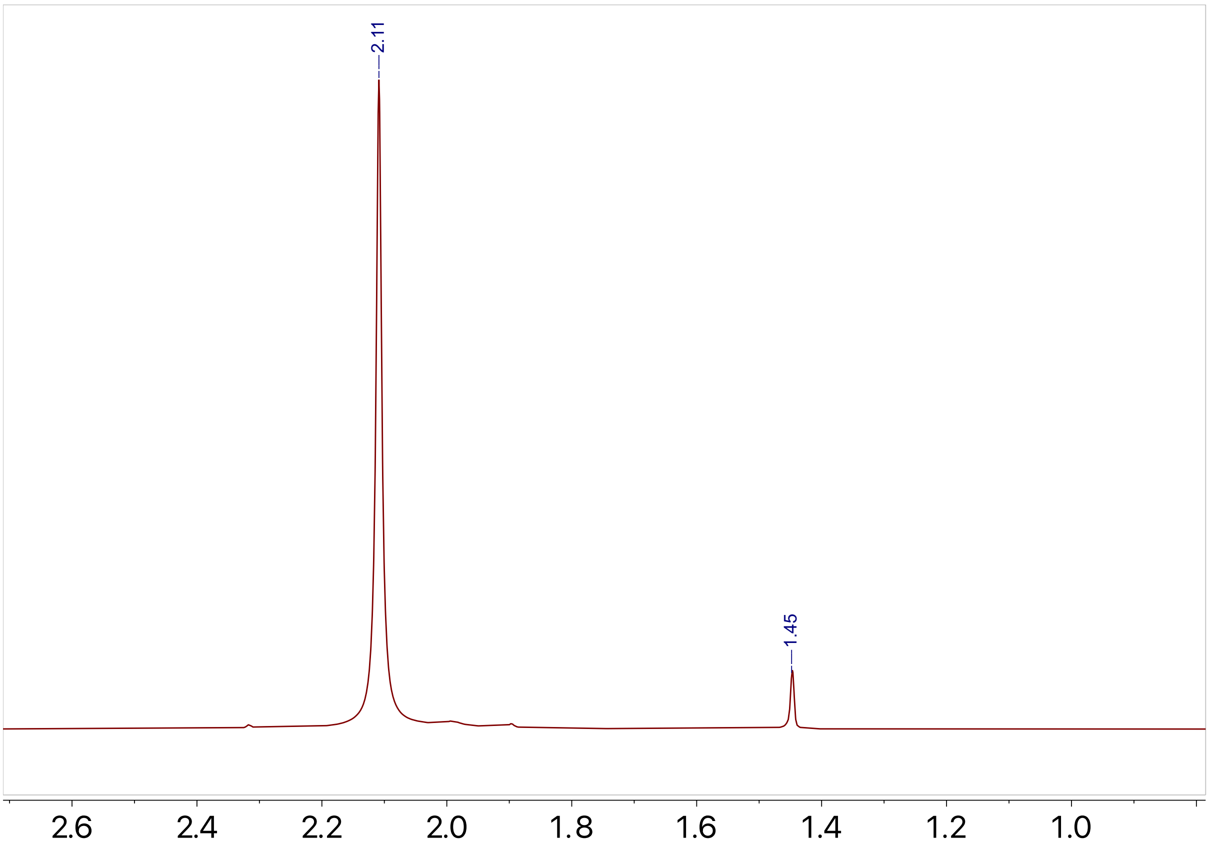

Figure S17: Evans method of 16.4 mM solution of complex **3** in C_6D_6 with toluene as internal standard at 299 K, zoomed in on the CH_3 signal of the toluene (internal standard).

3. Catalytic Details

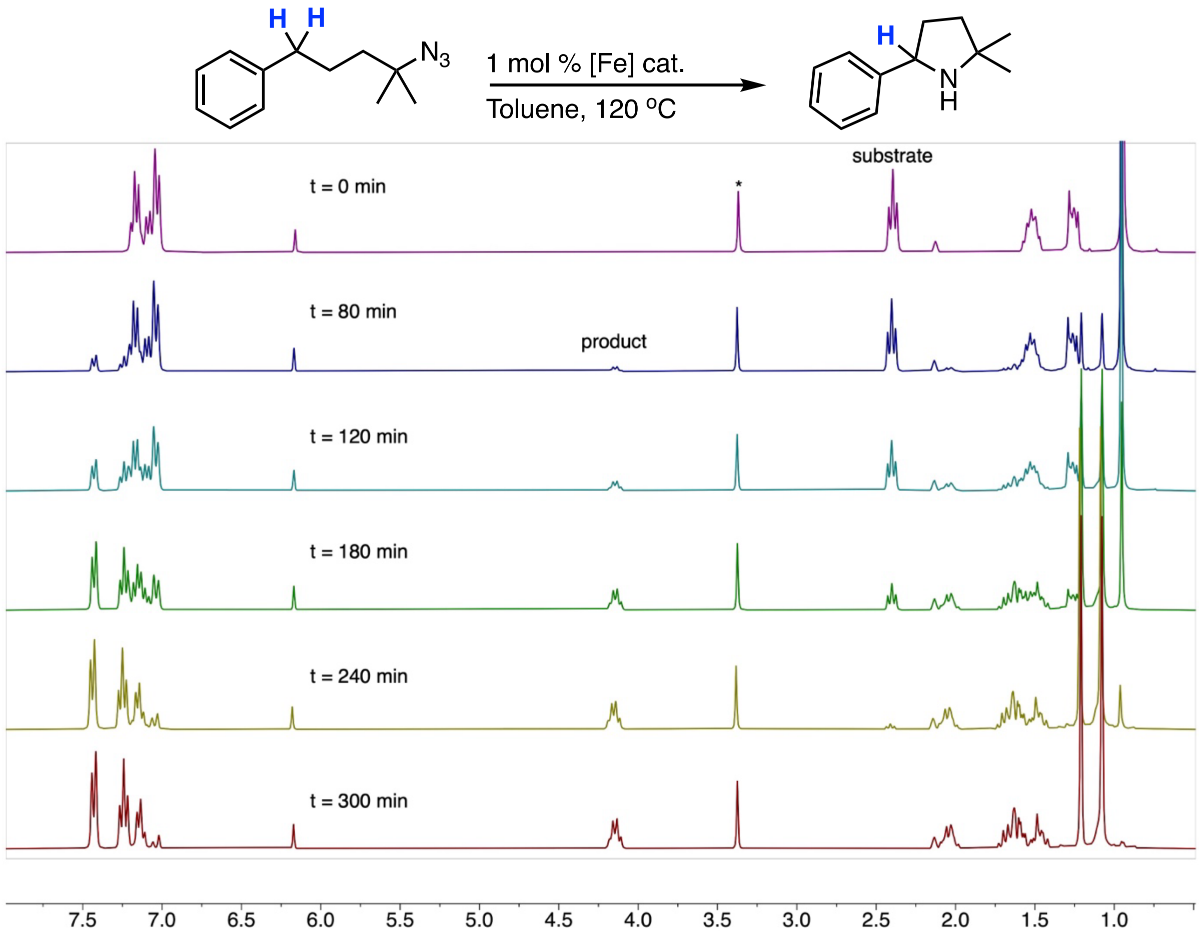

Figure S18: Stacked ^{1}H NMR spectra of a typical catalytic run with substrate **4** in toluene-d_8 (total volume = 0.55 mL). Catalytic conditions: 1 mol% catalyst loading ([**3**] = 4.48 mM) and $[sub]_0$ = 447.2 mM. Hydrogen atoms highlighted in blue refer to the signals for the substrate and product. The signal marked by (*) refers to the internal standard 1,3,5-trimethoxybenzene.

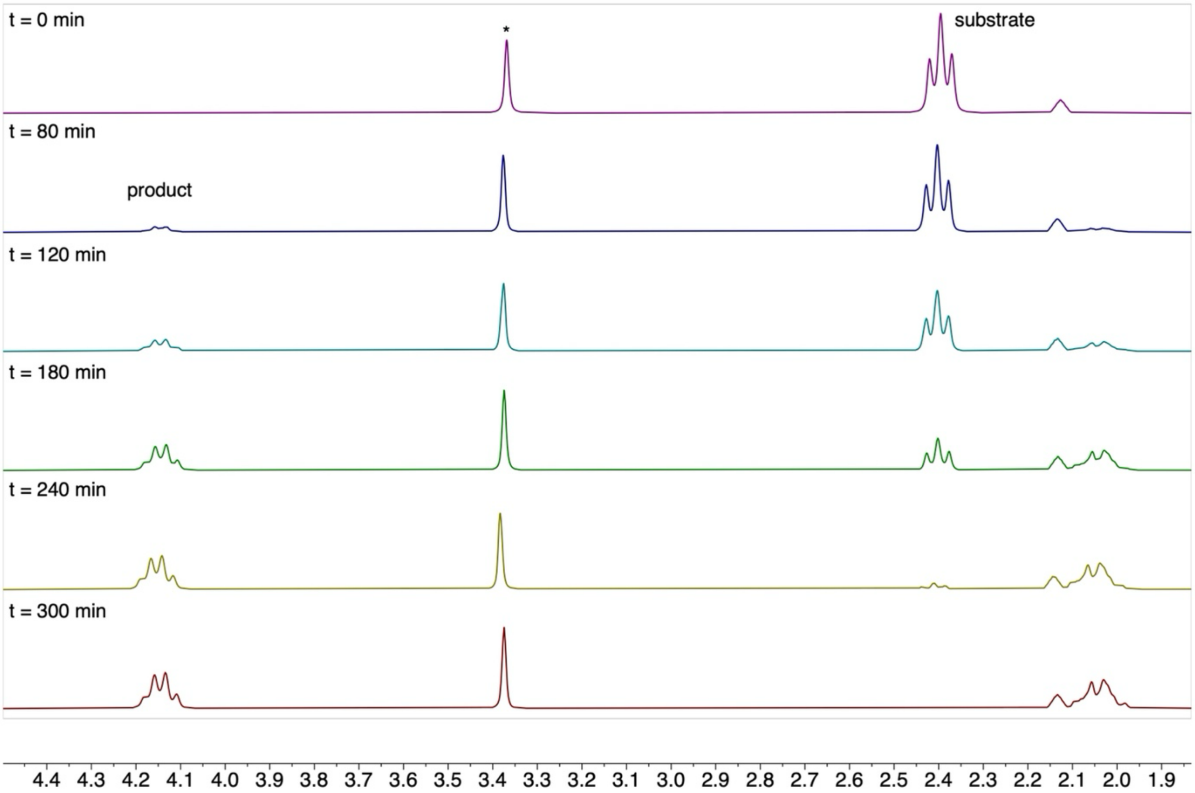

Figure S19: Zoom of the stacked plots (*cf* Fig. S18), highlighting the signals relevant to determine the yield of the reaction (* marks internal standard).

Complex 2 Kinetic Data

*Variable concentrations of complex **2**, fixed substrate concentration*

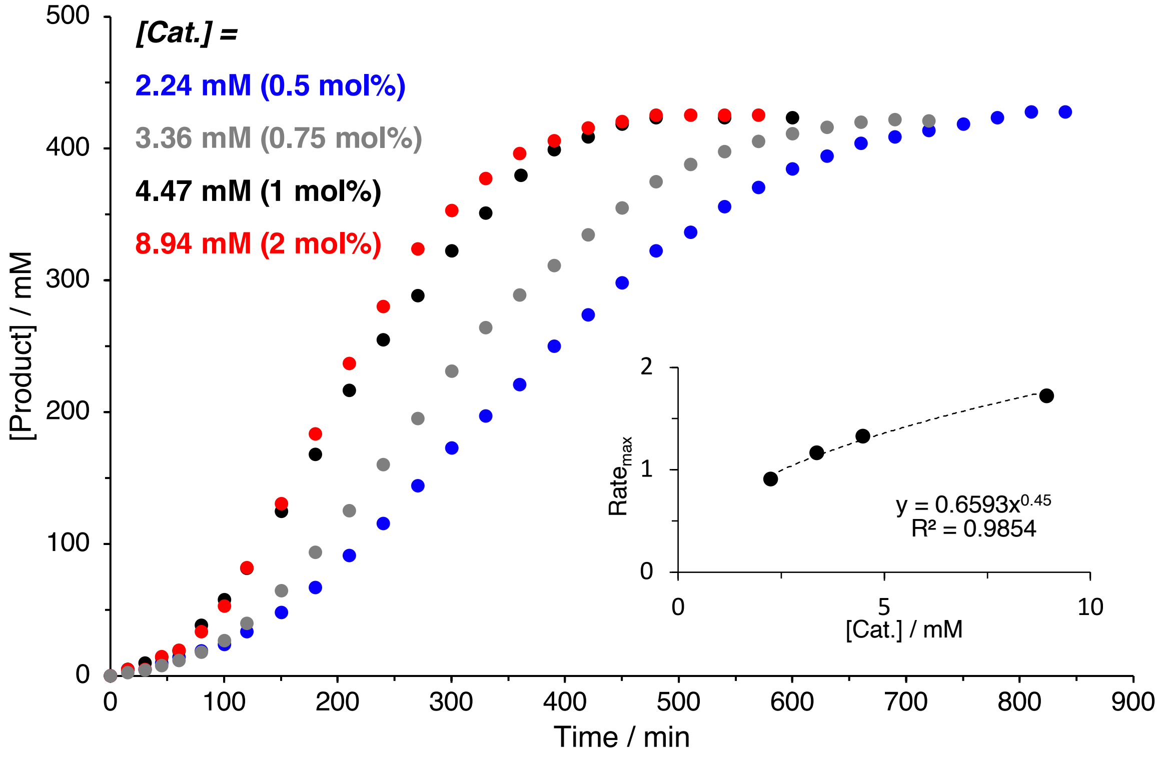

Figure S20: Formation of C–H aminated product over time with complex **2** at varying catalyst concentrations ([cat] = 2.24 – 8.94 mM with $[sub]_0$ = 447 mM). Product quantity was determined via ^{1}H NMR spectroscopy with reference to 1,3,5-trimethoxybenzene as an internal standard. Kinetic order with respect to catalyst was determined by calculating the maximum reaction rate from the reaction profile and plotting this against [catalyst], see Fig S21.

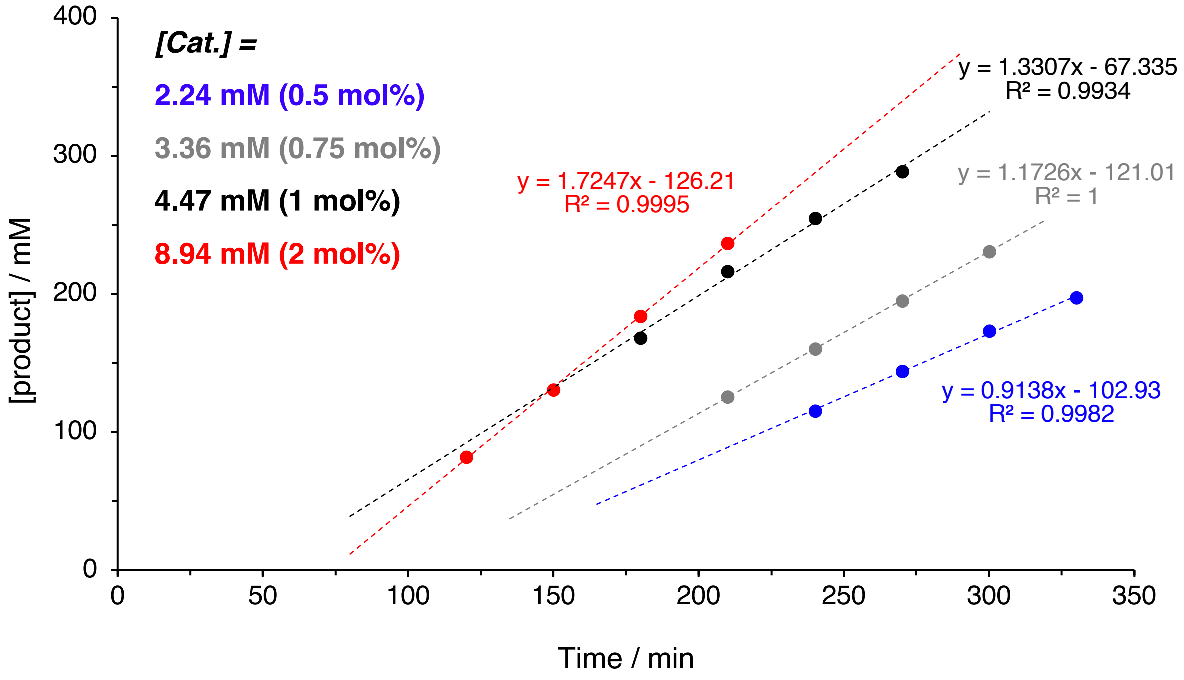

Figure S21: Formation of the C–H aminated product over time corresponding to the fastest reaction rate in the linear regime for complex **2** with varying catalyst concentrations ([cat] = 2.24 – 8.94 mM) with $[sub]_0$ = 447 mM.

*Fixed concentration of complex **2**, variable substrate concentrations*

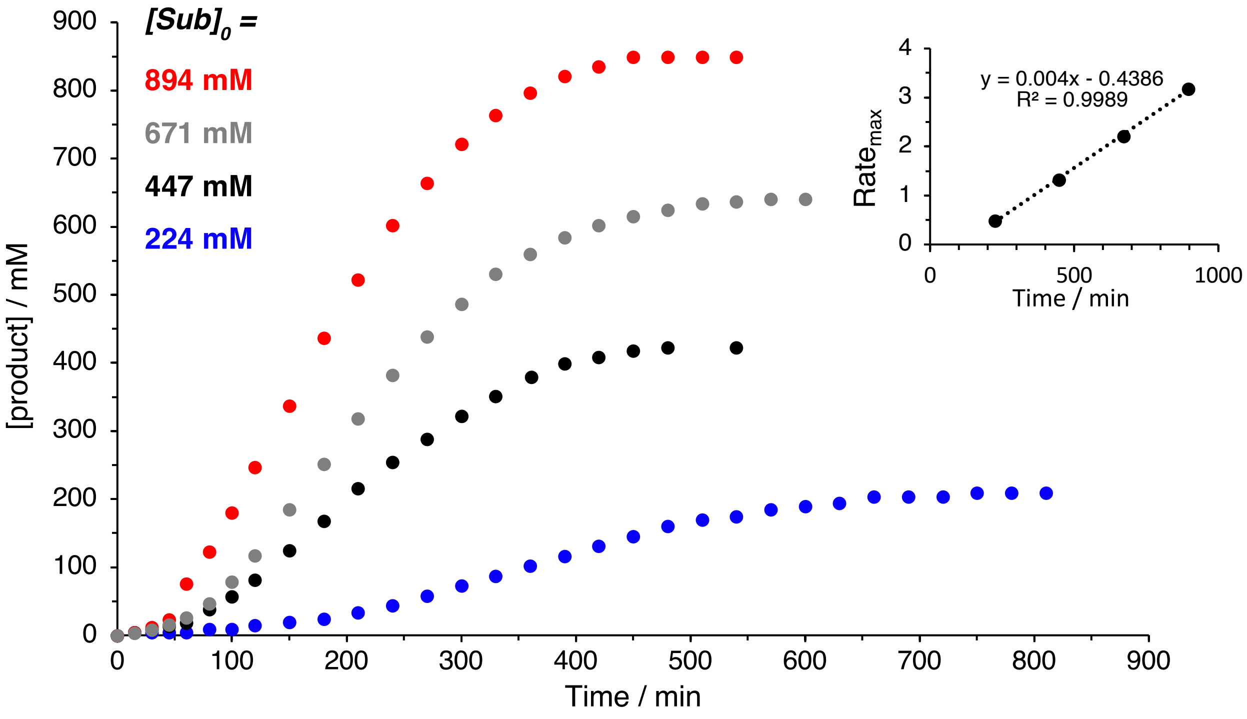

Figure S22: Formation of C–H aminated product over time with complex **2** at varying initial substrate concentrations ($[sub]_0$ = 224 – 894 mM with [cat] = 4.47 mM). Product quantity was determined via ^{1}H NMR spectroscopy with reference to 1,3,5-trimethoxybenzene as an internal standard. Kinetic order with respect to substrate was determined by calculating the maximum reaction rate from the reaction profile and plotting this against [catalyst], see Fig. S23.

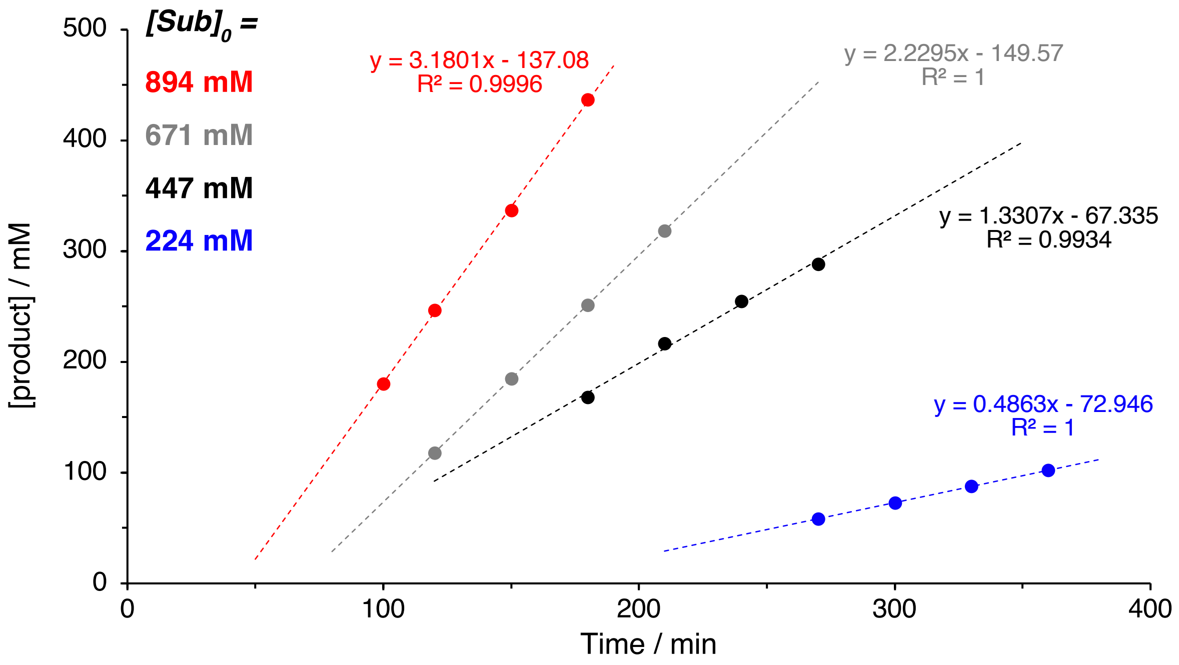

Figure S23: Formation of the C–H aminated product over time corresponding to the fastest reaction rate in the linear regime for complex **2** with varying initial substrate concentrations ($[sub]_0$ = 224 – 894 mM) with [cat] = 4.47 mM.

Variable Time Normalisation Analysis

As an alternative method for elucidating reaction orders, variable time normalisation analysis (VTNA)[S2] was performed on the kinetic data for complex **2** (Fig. S24–S28). The data was cut after the first data point with yield >75% to remove data points beyond the linear regime, in which decreasing substrate concentrations lead to a decrease in reaction rate.

reaction order in catalyst

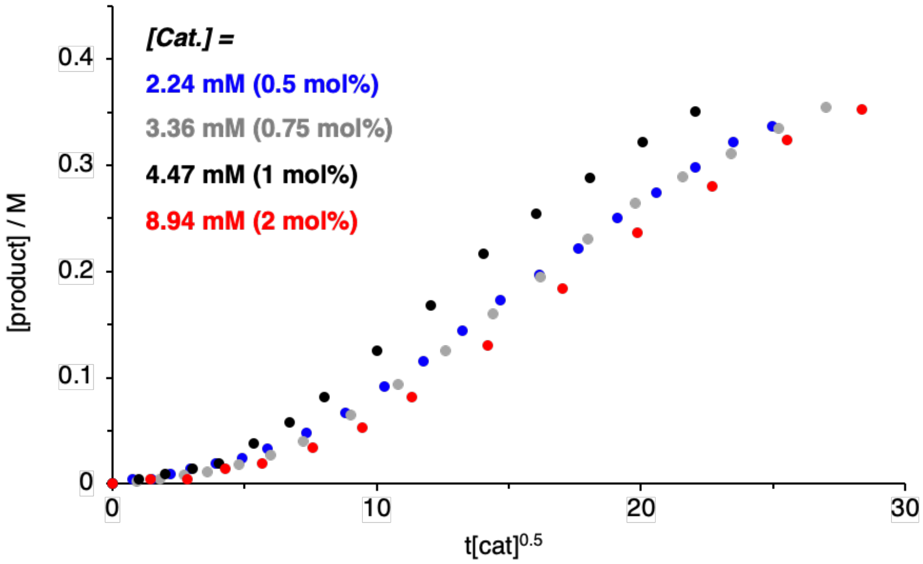

Figure S24: Selected area of the time conversion plot with product (M) and [Cat.] normalised against time, assuming 0.5 order.

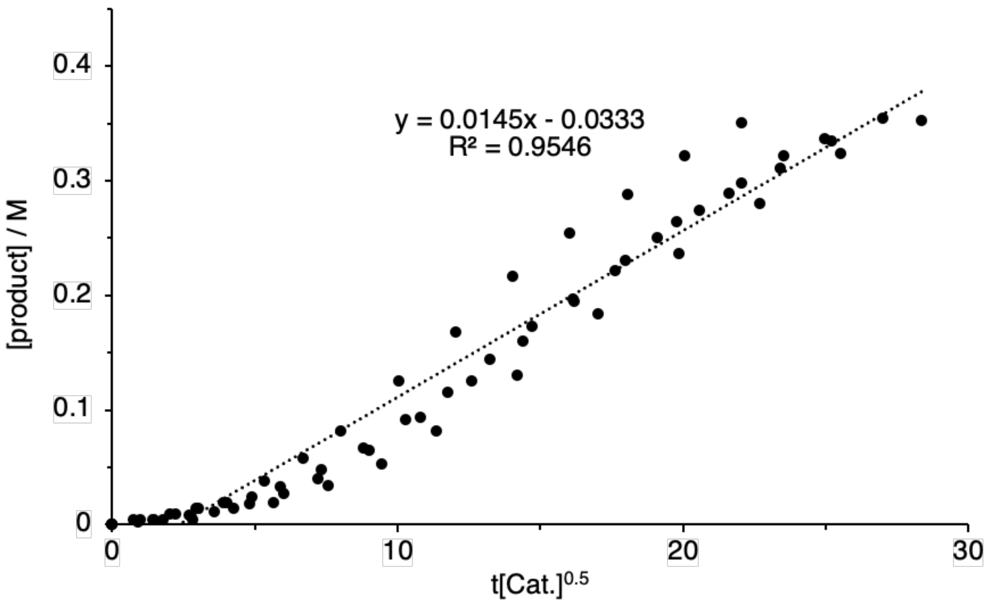

Figure S25: Linear regression of the points from Fig. S24 shows a strong correlation (R^2 = 0.9546)

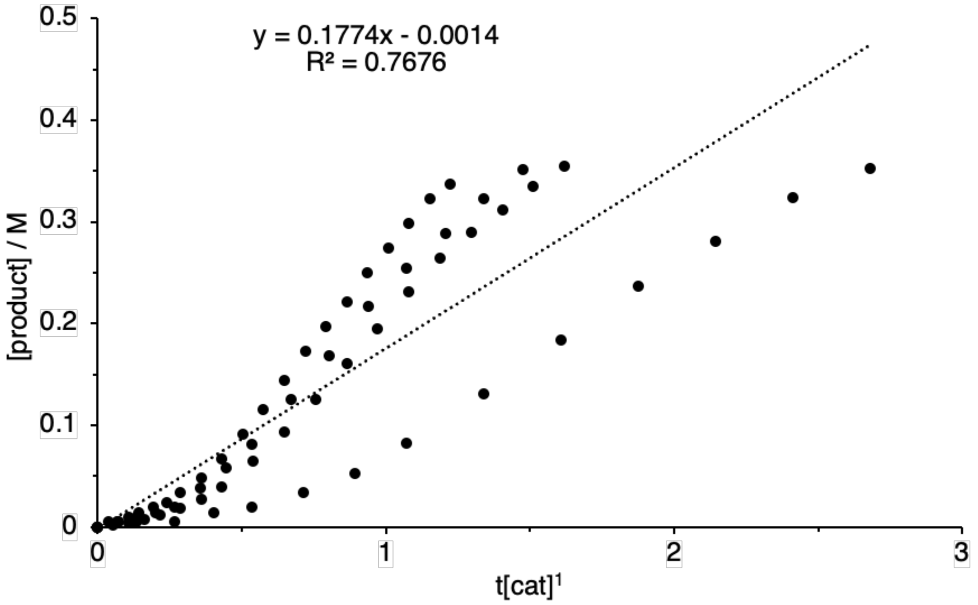

Figure S26: Linear regression of the same data for complex **2** assuming a first-order kinetic relationship, the regression is poor (R^2 = 0.7676).

*VTNA Method, Complex **2**, Substrate Order.*

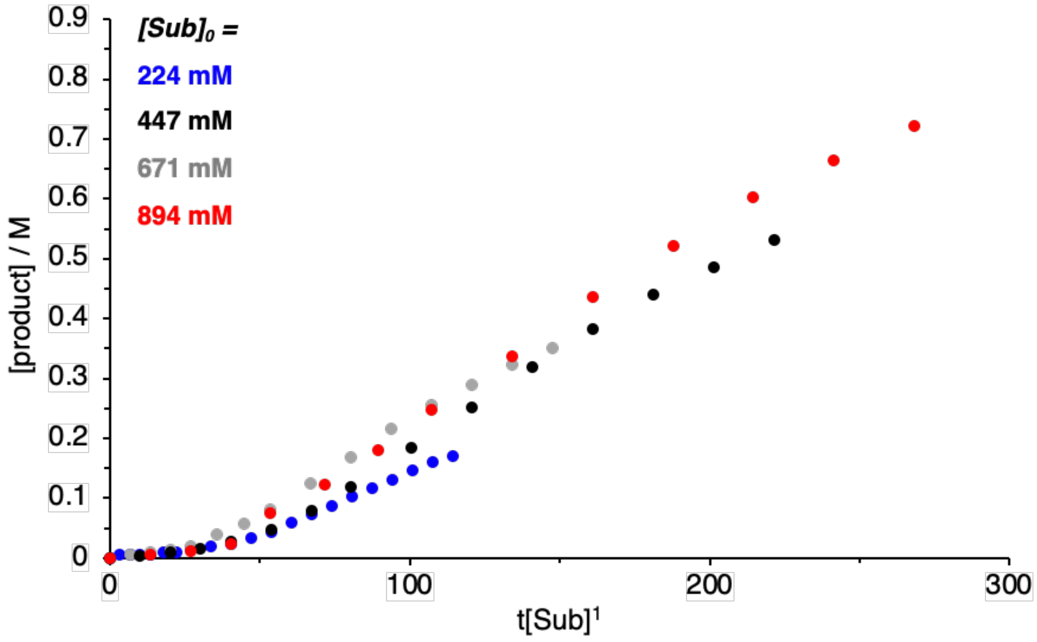

Figure S27: Selected area of the time conversion plot with complex **2** with product (M) and [Sub] normalised against time assuming 1st order in substrate.

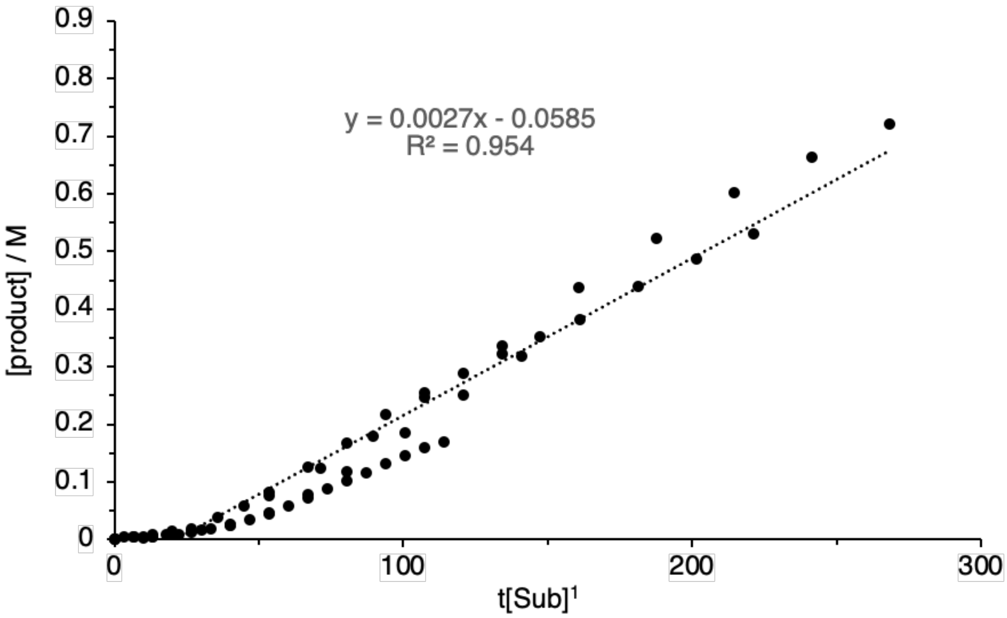

Figure S28: Linear regression of the points from Fig. S27 shows a strong correlation (R^2 = 0.954)

Complex 3 Kinetic Data

Variable concentrations of complex 3, fixed substrate concentration

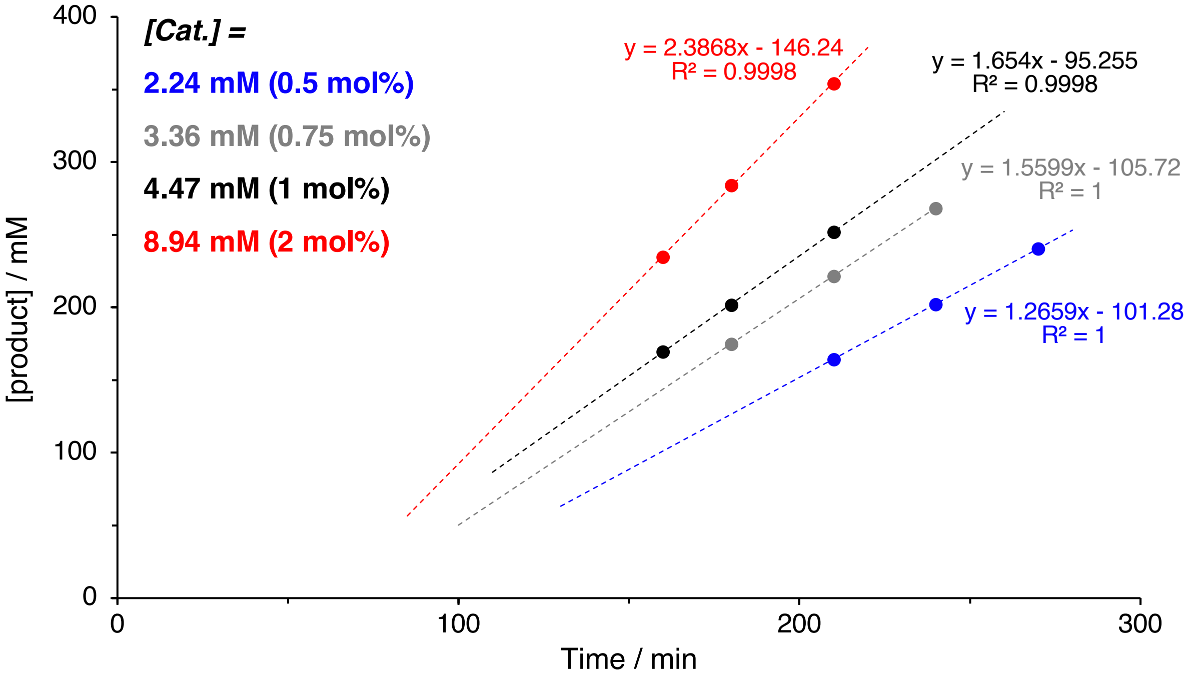

Figure S29: Formation of the C–H aminated product over time corresponding to the fastest reaction rate in the linear regime for complex **3** with varying catalyst concentrations ([cat] = 2.24 – 8.94 mM) with $[sub]_0$ = 447 mM.

*Fixed concentration of complex **3**, variable substrate concentrations*

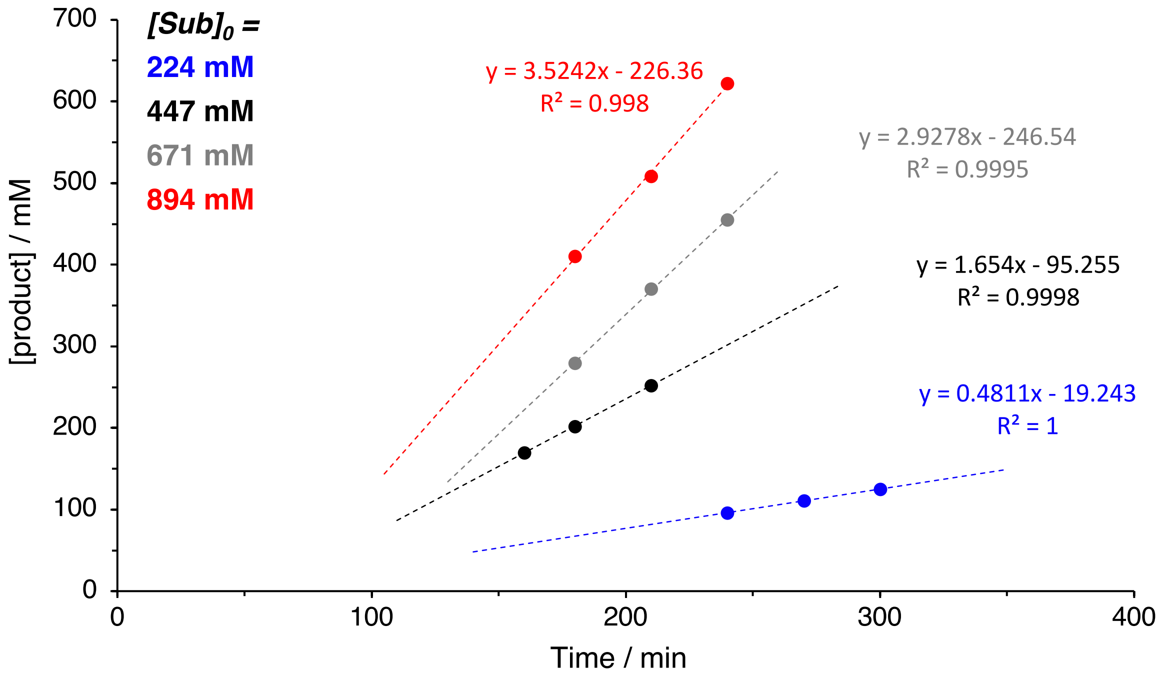

Figure S30: Formation of the C–H aminated product over time corresponding to the fastest reaction rate in the linear regime for complex **3** with varying initial substrate concentrations ($[sub]_0$ = 224 – 894 mM) with [cat] = 4.47 mM.

4. Crystallographic Details

Crystal structure determination. A suitable crystal of **2** or **3** was immersed in parabar oil and mounted at ambient conditions, and then transferred into the stream of nitrogen (173 K). All measurements were made on a *RIGAKU XtaLAB Synergy R*, HyPix-Arc 100 area-detector diffractometer[3] using mirror optics monochromated Mo *K*α radiation (λ = 0.71073 Å). The unit cell constants and an orientation matrix for data collection were obtained from a least-squares refinement of the setting angles of reflections in the range 2.127° < θ < 33.161° (**2**) and 2.324° < θ < 33.701° (**3**), respectively.

Data reduction was performed using the *CrysAlisPro*[3] program. The intensities were corrected for Lorentz and polarization effects, and a numerical absorption correction based on gaussian integration over a multifaceted crystal model with additional empirical absorption correction using spherical harmonics using SCALE3 ABSPACK in *CrysAlisPro*[3] was applied. Data collection and refinement parameters are given in Table S1.

The structures were solved by intrinsic phasing using *SHELXT*[4], which revealed the positions of all non-hydrogen atoms of the title compound. All non-hydrogen atoms were refined anisotropically. H-atoms were assigned in geometrically calculated positions and refined using a riding model where each H-atom was assigned a fixed isotropic displacement parameter with a value equal to 1.2Ueq of its parent atom (1.5Ueq for methyl groups).

Refinement of the structure was carried out on F^2 using full-matrix least-squares procedures, which minimized the function $\Sigma w(F_o^2 - F_c^2)^2$. The weighting scheme was based on counting statistics and included a factor to downweight the intense reflections. All calculations were performed using the *SHELXL-2014/7*[5] program in OLEX2.[6]

In the structure of **2**, twinning was detected where the second component corresponds to a rotation of -179.9825 deg around 1.0000 0.0002 0.0002 (reciprocal space), or 0.9977 0.0002 0.0672 (direct space), with a volume fractional contribution of 0.3354(5). The refinement was performed against the reflection file containing merged reflections of both components on a hkl 5 format.

Table S1. Crystallographic details for complexes 2 and 3

	**2**	**3**
CCDC Number	2361237	2361238
Empirical formula	$C_{42}H_{48}FeN_6O_2$	$C_{38}H_{44}FeN_6O_2$
Formula weight	724.71	672.64
Temperature (K)	173.01(10)	173.00(10)
Crystal system	monoclinic	monoclinic
Space group	$P2_1/n$	$P2_1/c$
a (Å)	10.1907(2)	12.26012(12)
b (Å)	24.2458(4)	34.8934(3)
c (Å)	15.4249(3)	8.50505(8)
α (°)	90	90
β (°)	95.8494(19)	100.1610(8)
γ (°)	90	90
Volume ($Å^3$)	3791.39(13)	3581.37(6)
Z	4	4
P_{calc} (g/cm^3)	1.270	1.248
μ (mm^{-1})	0.442	0.462
F (000)	1536.0	1424.0
Crystal size (mm^3)	0.156 × 0.096 × 0.087	0.157 × 0.151 × 0.116
Radiation	Mo Kα (λ = 0.71073)	Mo Kα (λ = 0.71073)
2θ for data collection	4.282 to 61.054	4.67 to 49.24
Index ranges	-14 ≤ h ≤ 14, -34 ≤ k ≤ 34, -21 ≤ l ≤ 21	-14 ≤ h ≤ 14, -40 ≤ k ≤ 40, -9 ≤ l ≤ 9
Reflections collected	21589	152558
Independent reflections	21589 [R_{int} = ?, R_{sigma} = 0.0405]	6014 [R_{int} = 0.0240, R_{sigma} = 0.0068]
Data/restraints/parameters	21589/0/471	6014/0/426
Goodness-of-fit on F^2	1.028	1.035
Final R indexes [I ≥2σ(I)]	R_1 = 0.0415, wR_2 = 0.0936	R_1 = 0.0254, wR_2 = 0.0655
Final R indexes [all data]	R_1 = 0.0655, wR_2 = 0.0991	R_1 = 0.0263, wR_2 = 0.0660
Largest diff. peak/hole (e $Å^3$)	0.34/-0.30	0.24/-0.27

5. References

S1 D. F. Evans, *J. Chem. Soc.*, 1959, 2003–2005.

S2 J. Burés, *Angew. Chem. Int. Ed.*, 2016, **55**, 16084–16087.

S3 Oxford Diffraction (2018). CrysAlisPro (Version 1.171.40.37a). Oxford Diffraction Ltd., Yarnton, Oxfordshire, UK.

S4 G. M. Sheldrick, *Acta Cryst C*, 2015, **A71**, 3–8.

S5 G. M. Sheldrick, *Acta Cryst C*, 2015, **C71**, 3–8.

S6 O. V. Dolomanov, L. J. Bourhis, R. J. Gildea, J. a. K. Howard and H. Puschmann, *J Appl Cryst*, 2009, **42**, 339–341.
